# Quantifying variant contributions in cystic kidney disease using national-scale whole-genome sequencing

**DOI:** 10.1172/JCI181467

**Published:** 2024-08-27

**Authors:** Omid Sadeghi-Alavijeh, Melanie M.Y. Chan, Gabriel T. Doctor, Catalin D. Voinescu, Alexander Stuckey, Athanasios Kousathanas, Alexander T. Ho, Horia C. Stanescu, Detlef Bockenhauer, Richard N. Sandford, Adam P. Levine, Daniel P. Gale

**Affiliations:** 1 Centre for Kidney and Bladder Health, University College London, London, United Kingdom.; 2 Genomics England, Queen Mary University of London, London, United Kingdom.; 3 Research Department of Pathology, University College London, London, United Kingdom.; 4 University Hospital and Katholic University Leuven, Leuven, Belgium.; 5 Academic Department of Medical Genetics, Cambridge University, Cambridge, United Kingdom.

**Keywords:** Genetics, Nephrology, Bioinformatics, Genetic diseases, Molecular genetics

## Abstract

**BACKGROUND:**

Cystic kidney disease (CyKD) is a predominantly familial disease in which gene discovery has been led by family-based and candidate gene studies, an approach that is susceptible to ascertainment and other biases.

**METHODS:**

Using whole-genome sequencing data from 1,209 cases and 26,096 ancestry-matched controls participating in the 100,000 Genomes Project, we adopted hypothesis-free approaches to generate quantitative estimates of disease risk for each genetic contributor to CyKD, across genes, variant types and allelic frequencies.

**RESULTS:**

In 82.3% of cases, a qualifying potentially disease-causing rare variant in an established gene was found. There was an enrichment of rare coding, splicing, and structural variants in known CyKD genes, with statistically significant gene-based signals in *COL4A3* and (monoallelic) *PKHD1*. Quantification of disease risk for each gene (with replication in the separate UK Biobank study) revealed substantially lower risk associated with genes more recently associated with autosomal dominant polycystic kidney disease, with odds ratios for some below what might usually be regarded as necessary for classical Mendelian inheritance. Meta-analysis of common variants did not reveal significant associations, but suggested this category of variation contributes 3%–9% to the heritability of CyKD across European ancestries.

**CONCLUSION:**

By providing unbiased quantification of risk effects per gene, this research suggests that not all rare variant genetic contributors to CyKD are equally likely to manifest as a Mendelian trait in families. This information may inform genetic testing and counseling in the clinic.

## Abstract 2

This research challenges traditional definitions of monogenic diseases by demonstrating varied genetic risk factors for cystic kidney disease, suggesting a complex interplay beyond simple Mendelian inheritance.

## Graphical abstract

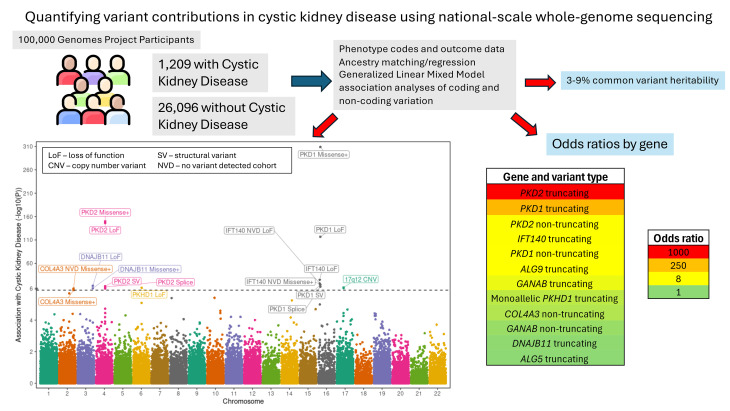

## Introduction

Cystic kidney disease (CyKD) is a term for any disease that causes multiple fluid-filled cysts within the kidney (excluding cystic degeneration of chronically diseased/failed kidneys). The term polycystic kidney disease (PKD) generally refers to CyKD in which the kidneys become enlarged and replaced by cysts and is almost always inherited as a Mendelian trait (either autosomal dominant or autosomal recessive). Ninety percent of CyKD is caused by autosomal dominant PKD (ADPKD), which accounts for approximately 10% of all patients receiving kidney replacement therapy for kidney failure (KF) in the United Kingdom (UK) (1). It is estimated that between 1 in 800 and 1 in 1,000 of the population has ADPKD (2), making this the commonest monogenic cause of life-shortening disease worldwide.

Rare monoallelic variants in 2 genes, *PKD1* and *PKD2*, cause the majority of ADPKD, while biallelic variants in *PKHD1* cause the majority of autosomal recessive PKD (ARPKD). *PKD1* accounts for approximately 80% of ADPKD diagnoses, while *PKD2* accounts for approximately 15% (2). *PKD1* encodes polycystin-1 (PC1), a large multidomain glycoprotein, while *PKD2* encodes polycstin-2 (PC2), which is a nonspecific cation channel that interacts with PC1. Both proteins are found in primary cilia and play a role in mechanotransduction, transferring external information to the cell. While the function of PC1, PC2, and the PC1-PC2 complex is still not fully understood, it is increasingly accepted that these proteins prevent cystogenesis via a dose-dependent mechanism (3). *PKHD1* encodes the fibrocystin protein and acts via a common polycystin pathway to cause cysts in ARPKD usually manifesting at a younger age than ADPKD and associated with extrarenal features, including liver fibrosis (4).

There has been increasing recognition of the genetic heterogeneity of CyKD, including contributions of genes other than *PKD1/PKD2*. Among the 10%–15% of patients suspected to have ADPKD who have no explaining variants in either *PKD1* or *PKD2*, next-generation sequencing (NGS) has identified several other genes in which variants cause ADPKD, including *DNAJB11* (5), *GANAB* (6), *ALG9* (7), and more recently, *IFT140* (8) and *ALG5* (9). Presently, a clinical diagnosis of PKD implies a high risk of kidney failure, but pathogenic variants in these more recently described genes seem to carry a lower penetrance, manifesting as sporadic or non-Mendelian disease in individuals or families, with a less severe phenotype. To date, estimates of penetrance and outcomes with these rarer genetic variants are based predominantly on case series and reports of individual families, increasing the risk of ascertainment bias because more severely affected families are more likely to have come to light. Providing accurate data about the adverse consequences of these rarer variants is essential to prognostication, informing life and reproductive choices for patients and family members.

Obtaining a molecular diagnosis for CyKD can also inform choice of therapy (10, 11). Genetic testing for *PKD1* variants is challenging, owing to its high GC content, numerous repetitive regions, and the presence of 6 pseudogenes that share 97% of their sequence with *PKD1* (12, 13). This has traditionally necessitated the use of Sanger sequencing of a long-range PCR amplification product (13), a method that is technically difficult and expensive. Whole-genome sequencing (WGS) has been shown to offer high-quality *PKD1* sequencing (14). WGS provides more uniform coverage and avoids capture bias, resulting in increased sensitivity to detect structural variants (SVs) and rare single nucleotide variants (SNVs) compared with exome (or capture-based) sequencing platforms, even within regions targeted by the latter (15, 16). In addition, WGS benefits from economies of scale and the associated costs have dropped dramatically over the past decade (17). However, few studies have looked at the utility of WGS in CyKD on a large scale (18).

Large-scale WGS data sets facilitate the assessment of the association of variants across the allelic spectrum with a certain phenotype in an unbiased genome-wide manner. This is especially useful in the study of the genomics of rare diseases, where approaches reliant on the sequencing of candidate gene panels or single genes of interest lead to discovery bias. In this study, we perform genome-wide association analyses using WGS data from 1,209 CyKD cases and 26,096 ancestry-matched controls. We perform variant-level, gene-level, pathway-level, and time-to-event (TTE) association analyses, including rare variants, common variants, and SVs in a diverse-ancestry population, providing the largest unbiased assessment of CyKD to date. We identify strong associations in known (*PKD1*, *PKD2*, *DNAJB11*, and *IFT140*) and potentially novel genes (*COL4A3,* monoallelic *PKHD1*) across various variant types. We leverage these data to conduct the largest common variant genome-wide association study (GWAS) of CyKD to date, showing no significant associations, and use this to define the common variant heritability of CyKD as between 3% and 9%. This approach provides a comprehensive, unbiased framework for large-scale WGS analysis that can be utilized to gain insights into the molecular mechanisms underlying other rare diseases going forward.

## Results

**Figure 1:**
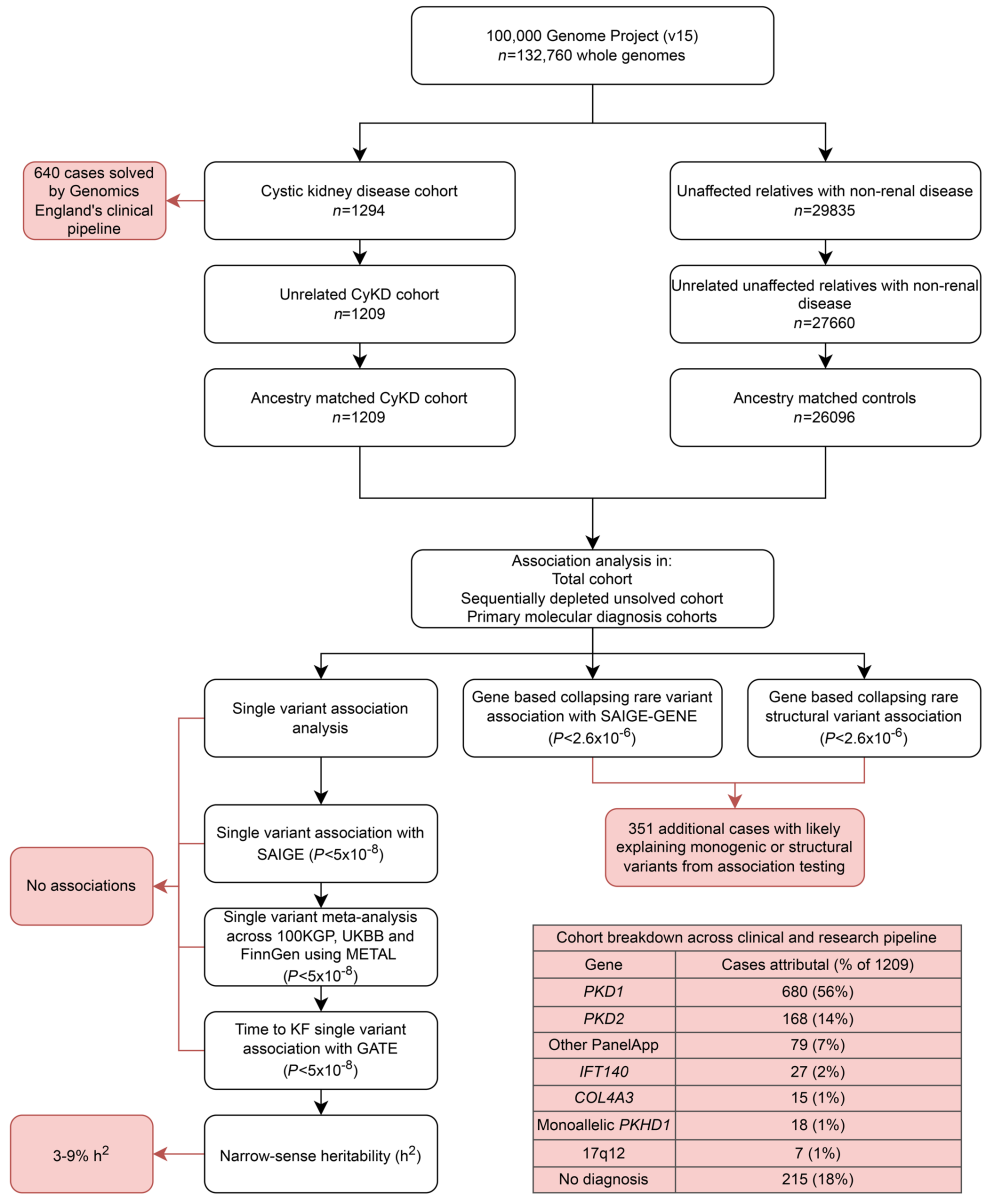
Study workflow detailing high-level methods and results. Flow diagram depicting the methods and high-level results from the study. CyKD, cystic kidney disease; 100KGP, 100,00 genome project; UKBB, UK Biobank, GATE, genetic analysis of time to event.

A full study workflow with high-level results can be found in Figure 1.

### The CyKD cohort demographics.

The CyKD cohort consisted of 1,558 participants recruited to the 100,000 Genomes Project (100KGP), of which 1,294 were probands (full breakdown of recruited pedigrees in Supplemental Table 1A; supplemental material available online with this article; https://doi.org/10.1172/JCI181467DS1). The demographic information and top 5 most frequent Human Phenotype Ontology (HPO) codes of probands are shown in Table 1.

### The clinically validated arm of the 100KGP gave a molecular diagnosis to 53% of the CyKD cohort.

Of these probands, 1,290 had a genetic outcome from the 100KGP clinical pipeline: 640 (52.93%) were solved, 34 (2.81%) were partially solved, 79 (6.54%) had missing data, and 537 (44.42%) were unsolved. The top 3 molecular diagnoses were *PKD1*-truncating (340 [26%]), *PKD2*-truncating (122 [9.5%]), and *PKD1*-nontruncating (118 [9.1%]) variants. The full breakdown of solved cases and the types of variants can be found in Supplemental Table 2 (3 patients were solved for primary conditions unrelated to their CyKD, e.g., intellectual disability, and were not included in this table and 12 cases did not have a gene recorded despite being listed as solved).

### Outcome data show those with pathogenic PKD1 variants have the worst renal prognosis followed by those without a diagnosis.

**Figure 2:**
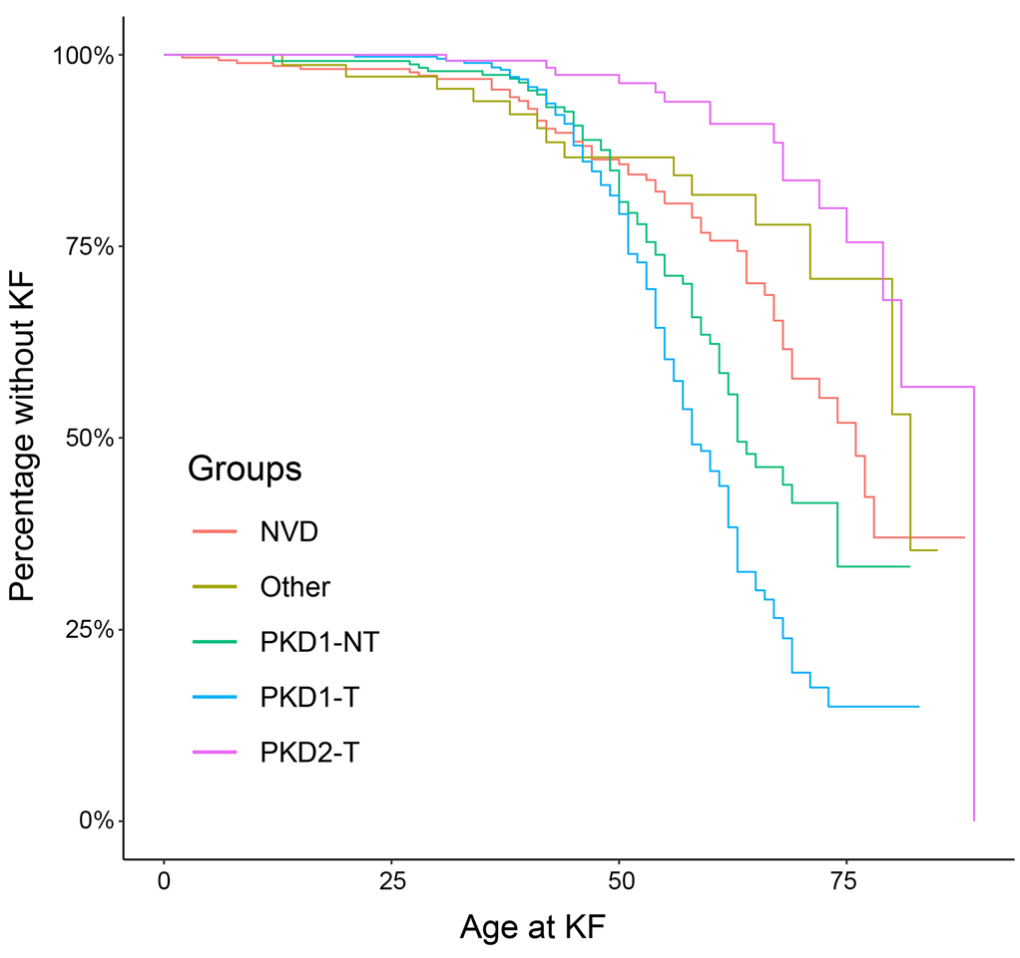
Outcome data show those with pathogenic *PKD1* variants have the worst renal prognosis followed by those without a diagnosis. Kaplan-Meier graph of kidney failure (KF) by variant type. PKD1-T, PKD1-truncating variant; PKD1-NT, PKD1-nontruncating variant; PKD2-T, PKD2-truncating variant; Other, another variant in the PanelApp cystic kidney disease gene panel.

Of the 1,290 cases, 578 (44.8%) had data regarding kidney function in the form of HPO (PMID: 37953324) or Hospital Episode Statistics (HES) codes, of whom 398 (68.9%) had reached KF (full breakdown in Supplemental Table 1, B and C). Survival analysis of these cohorts followed what is known about the renal prognosis of variants in CyKD (Figure 2).

### Combining the clinical and research pipelines identified a potential explaining genetic variant in the majority of CyKD cases.

Of the 1,209 ancestry-matched CyKD cases, 994 (82%) had a potentially explaining monogenic or single SV identified when combining the clinical pipeline results with those who had variants identified via collapsing-gene-based analyses (Supplemental Table 3). These are discussed in greater detail below. Of note, the research cohort consists of a subset of cases with ancestry-matched controls (*n* = 1,209).

### Unbiased rare variant analysis highlights IFT140 and COL4A3 as important genes involved in CyKD.

**Figure 3:**
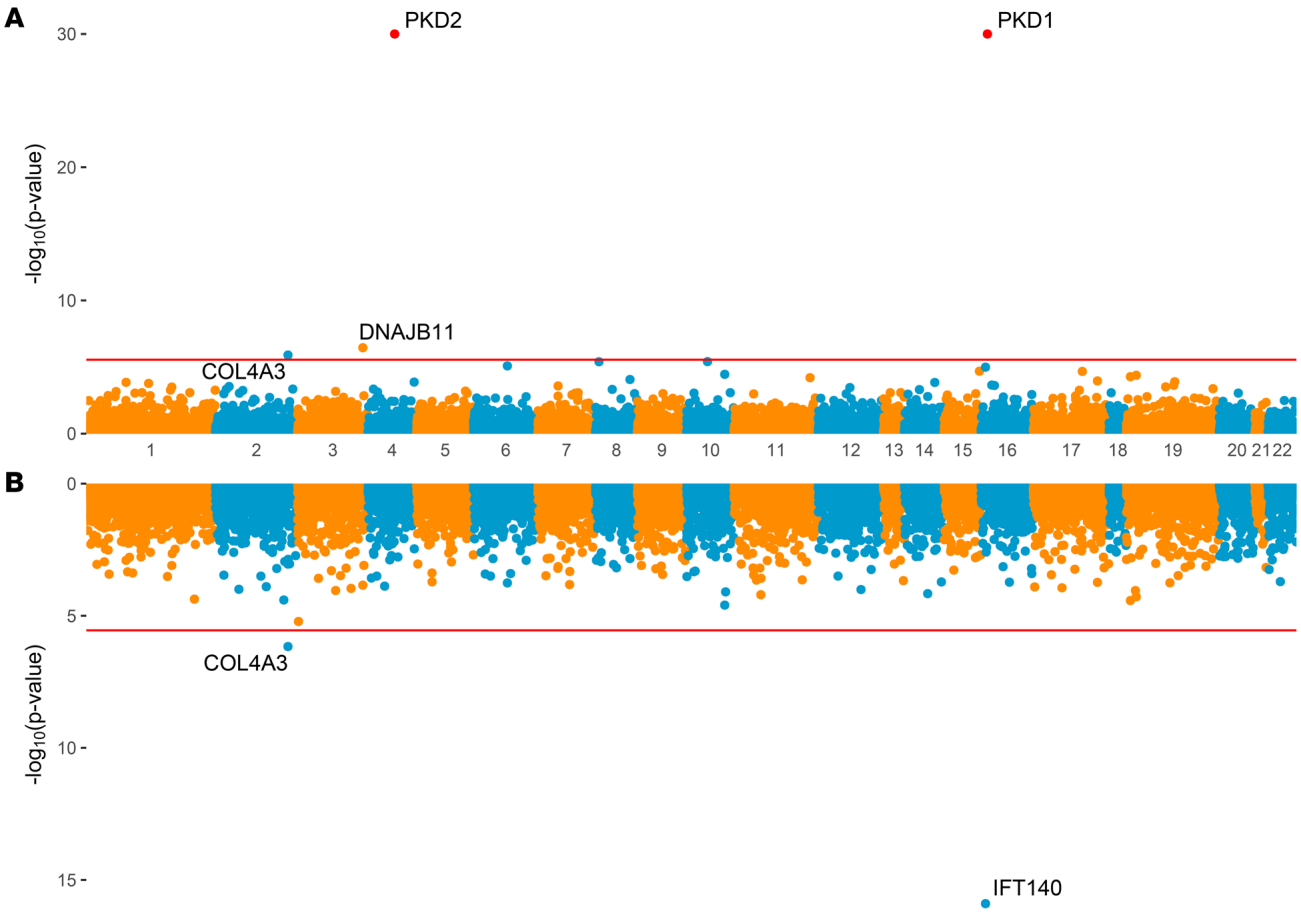
Unbiased rare variant analysis highlights *IFT140* and *COL4A3* as important genes involved in CyKD. Gene-based Miami plots of the SAIGE-GENE “missense+” analyses. Each data point is a gene, made up of variants that qualified under their respective mask. (**A**) Missense+ analysis of the total ancestry-matched cohort of 1,209 cases and 26,096 controls showing a significant enrichment of cases for *PKD1*, *PKD2*, *DNAJB11*, and *COL4A3*. *PKD1* and *PKD2* are highlighted, as the plot is capped at an association of 1 × 10^–30^. Actual associations: *PKD1* (*P* = 1.17 × 10^–309^), *PKD2* (*P* = 1.96 × 10^–150^). (**B**) Missense+ analysis of the depleted cohort of 308 cases versus 26,096 controls showing enrichment of cases for *IFT140* and *COL4A3*. The horizontal line indicates the threshold for exome-wide significance. Generalized logistic regression modeling, as implemented using optimal sequence kernel association testing (SKAT-O) via SAIGE-GENE, was used to generate these data.

Rare variant analysis of the ancestry-matched cohort of 1,209 cases and 26,096 ancestry-matched controls under the “missense+” mask showed a significant enrichment of cases for *PKD1* (*P* = 1.17 × 10^–309^, odds ratio [OR] 10.60, 95% CI 9.35–12.01), *PKD2* (*P* = 1.96 × 10^–150^, OR 19.07, 95% CI 15.13–23.99), *DNAJB11* (*P* = 3.52 × 10^–7^, OR 1.07, 95% CI 0.95–1.21), and *COL4A3* (*P* = 1.26 × 10^–6^, OR 3.02, 95% CI 2.10–4.22) (Figure 3A). There was no evidence of genomic inflation (λ < 1; see Q-Q plot in Supplemental Figure 3B).

Removing cases solved by 100KGP and patients that had a bioinformatically ascertained potentially disease-causing variant in a known cystic gene left 308 cases (at the time of analysis *IFT140* was not a known CyKD gene). Repeating the rare variant analysis under the “missense+” mask in this group showed a significant enrichment of cases with variants in *IFT140* (*P* = 1.26 × 10^–16^, OR 5.57, 95% CI 3.63–8.21) and *COL4A3* (*P* = 6.83 × 10^–7^, OR 4.93, 95% CI 2.77–8.11) compared with 26,096 controls (Figure 3B).

Using REVEL, a different annotation method for missense variants in the total and unsolved CyKD cohort, led to largely similar results but with smaller association values. In the total cohort, *PKD1* (*P* = 1.63 × 10^–35^, OR 22.41, 95% CI 14.57–34.49), *PKD2* (*P* = 3.84 × 10^–14^, OR 21.87, 95% CI 10.47–45.71), and *COL4A3* (*P* = 8.84 × 10^–6^, OR 3.42, 95% CI 2.04–5.47) remained significant with the loss of the *DNAJB11* signal. Of note, *PKHD1* (*P* = 8.12 × 10^–5^, OR 3.56, 95% CI 1.62–7.02) and *COL4A4* (*P* = 7.74 × 10^–4^, OR 3.52, 95% CI 1.80–6.42) increased their significance. In the unsolved CyKD cohort, *COL4A3* (*P* = 1.09 × 10^–6^, OR 6.50, 95% CI 3.01–12.50) remained significant (Supplemental Tables 6 and 8). Of note, other known genes causing CyKD such as *GANAB* and *ALG8* were not found to be enriched at a population level in this cohort. Analysis of a combined unsolved CyKD and polycystic liver disease cohort (*n* = 359) did not reveal any additional associated genes (Supplemental Tables 10 and 14).

### IFT140 and COL4A3 variants in unsolved individuals most likely represent their primary diagnosis.

Out of the 308 unsolved cases, 27 (8.8%) had a qualifying variant in *IFT140* under the “missense+” mask. Of the 27 cases, all were heterozygous for the qualifying variants. None of the variants individually reached genome-wide significance.

Analysis of SVs intersecting with *IFT140* revealed 2 additional cases (0.65%) with heterozygous exon-crossing SVs from the 308 unsolved cystic disease cases: one patient had a 3.6 kb deletion spanning exon 16 and 17 and another patient had 2 different inversions (12.6 kb and 8.1 kb). Four exon-crossing SVs (3 deletions and 1 tandem duplication) were seen in 4 different controls (0.015%). There was enrichment of *IFT140* SVs in cases versus controls (*P* = 0.0032), although this was not significant at the genome-wide level. None of the 27 initial cases with *IFT140* SNVs had detectable CNVs affecting *IFT140*, compared with 3 CNVs seen in 26,096 controls (*P* = 1).

There were no plausible second variants within *IFT140* or other known cystic kidney genes in any of these individuals. A full variant and phenotypic breakdown of the *IFT140* cases can be found in Supplemental Table 4A.

Among the 15 unsolved CyKD patients with qualifying variants in *COL4A3* under the “missense+” mask, all were heterozygous for their respective variants and did not overlap with the unsolved *IFT140* cohort listed above. None of the variants individually reached genome-wide significance. There was no plausible second variant in known cystic kidney genes that could explain the phenotype. Of note, 4 of the *COL4A3* patients had liver cysts. We reanalyzed these 4 patients, searching for known genetic causes of polycystic liver disease, but none were found. A full variant and phenotypic breakdown of the *COL4A3* cases can be found in Supplemental Table 4B.

### Analysis of loss-of-function (protein truncating) variants identifies monoallelic defects of PKHD1 in unsolved CyKD.

**Figure 4:**
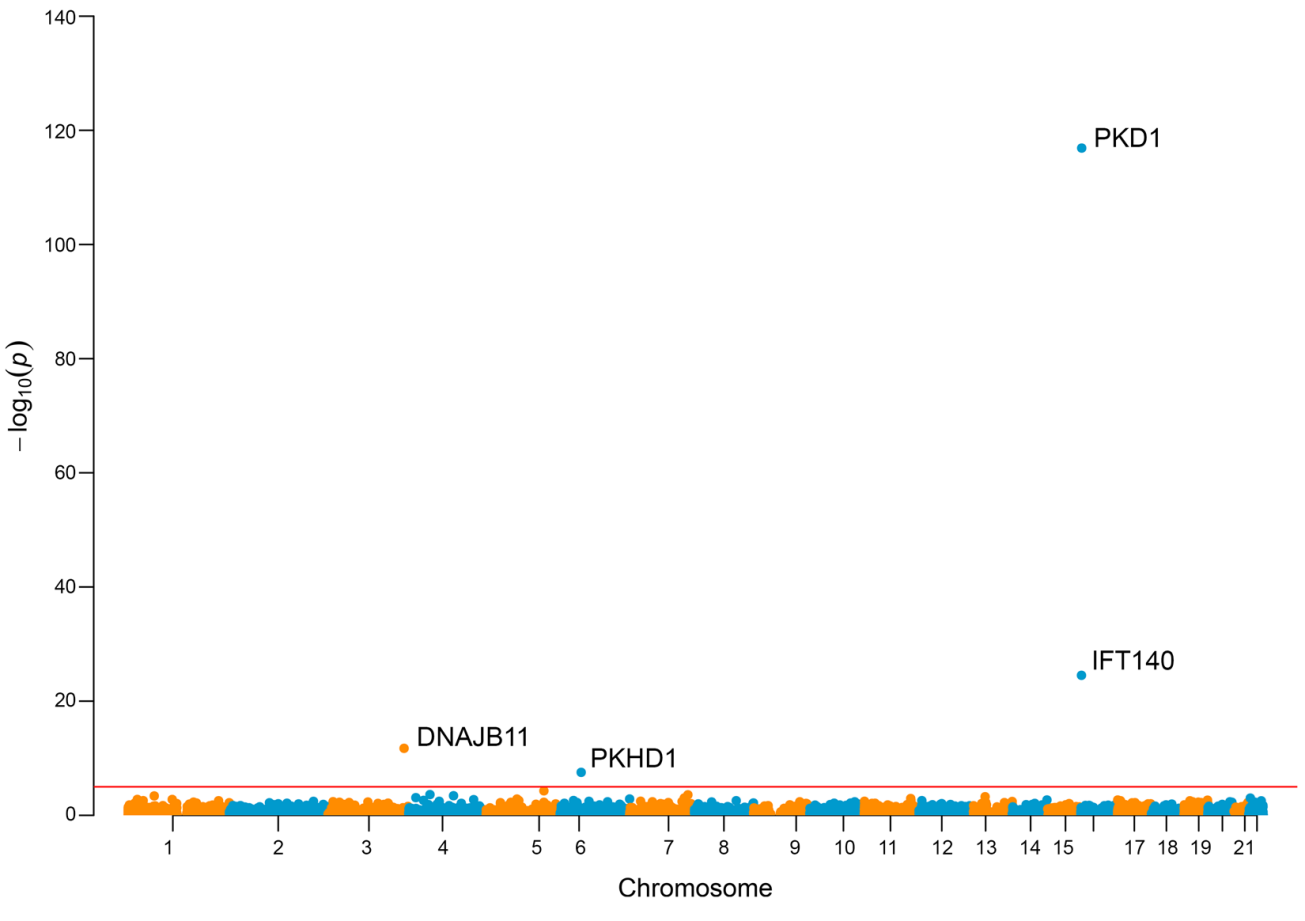
Analysis of loss-of-function (protein truncating) variants identify monoallelic defects of *PKHD1* in unsolved CyKD. Gene-based Manhattan plot of the SAIGE-GENE “loss-of-function” analysis of the total ancestry-matched cohort of 1,209 cases and 26,096 controls showing a significant enrichment of cases for *PKD1*, *PKD2*, *DNAJB11*, *IFT140*, and *PKHD1*. Each data point is a gene, made up of variants that qualified under their respective mask. The red line indicates an exome-wide significance level of *P* = 2.5 × 10^–6^. Generalized logistic regression modeling, as implemented using optimal sequence kernel association testing (SKAT-O) via SAIGE-GENE, was used to generate these data.

Collapsing rare variants that had a high confidence call for loss-of-function under the “LoF” mask (i.e., analysis restricted to protein length-altering variants, excluding all missense variants) revealed significant enrichment of cases for *PKD2* (*P* = 3.05 × 10^–147^, OR 130.85, 95% CI 83.66–215.37), *PKD1* (*P* = 1.29 × 10^–117^, OR 36.01, 95% CI 30.52–42.23), *IFT140* (*P* = 3.00 × 10^–25^, OR 14.03, 95% CI 7.91–24.45), *DNAJB11* (*P* = 1.84 × 10^–12^, OR 1.07, 95% CI 0.95–1.21), and *PKHD1* (*P* = 2.98 × 10^–08^, OR 4.07, 95%CI 2.24–6.88) (Figure 4).

Removing cases with qualifying variants in *IFT140* and *COL4A3* left 266 unsolved cases in whom rare variant testing did not reveal any additional significant associations (Supplemental Figure 3).

In all presented analyses, the patients were heterozygous for their qualifying variants, except in *DNAJB11* where 59 of the 369 cases that had qualifying variants within the “missense+” mask were homozygous.

There were 61 predicted LoF variants in *PKHD1* that made up the association signal in the LoF mask analysis of the whole CyKD cohort. These were seen in 50 cases, of which 22 were solved, 2 were partially solved, 24 were unsolved, and 2 were unascertainable. All 50 cases were heterozygous for the variant that made up the signal.

Of the 22 solved cases, 3 patients had a diagnosis of ARPKD secondary to biallelic *PKHD1* variants, and 19 had a diagnosis of ADPKD due to variants in *PKD1* or *PKD2*. In the 2 partially solved cases, both patients had a second *PKHD1* variant deemed to be a variant of unknown significance (VUS).

Of the 24 unsolved cases with a single LoF *PKHD1* variant, 4 also had a computationally predicted high-impact nontruncating variant in *PKD1*, and 1 (in addition to the *PKHD1* variant) had a predicted high-impact nontruncating *PKD2* variant.

In the remaining 18 cases with a single heterozygous *PKHD1* LoF variant, there were no SNVs or SVs that would imply compound heterozygosity (and a diagnosis of ARPKD), or potentially pathogenic variants in any other gene associated with CyKD. Two patients had a second *PKHD1* variant, with a combined annotation–dependent depletion (CADD) score of greater than 20 in *PKHD1*, but both had been deemed “likely benign” by Clinvar (Clinvar ID: 1187104 and 102305).

In total, 634 (2.4%) of the 26,096 controls carried qualifying monoallelic *PKHD1* LoF variants. When compared with the 18 (6.7%) out of 266 unsolved cases with no clear molecular diagnosis, there is a significant enrichment of *PKHD1* variants in the unexplained CyKD cohort (*P* = 5.85 × 10^–6^, OR 2.92, 95% CI 1.69–4.76). Three of the 18 monoallelic *PKHD1* cases had reached KF at a median age of 42 years. There was no statistically significant difference between the rates of liver cysts between the monoallelic *PKHD1* cohort and the general CyKD cohort (*P* = 0.31). The full demographic details of the *PKHD1* cohort can be found in Supplemental Table 4C.

### Noncoding collapsing analysis of the no-variant-detected cohort revealed enrichment of splice site variants in PKD1 and PKD2.

**Figure 5:**
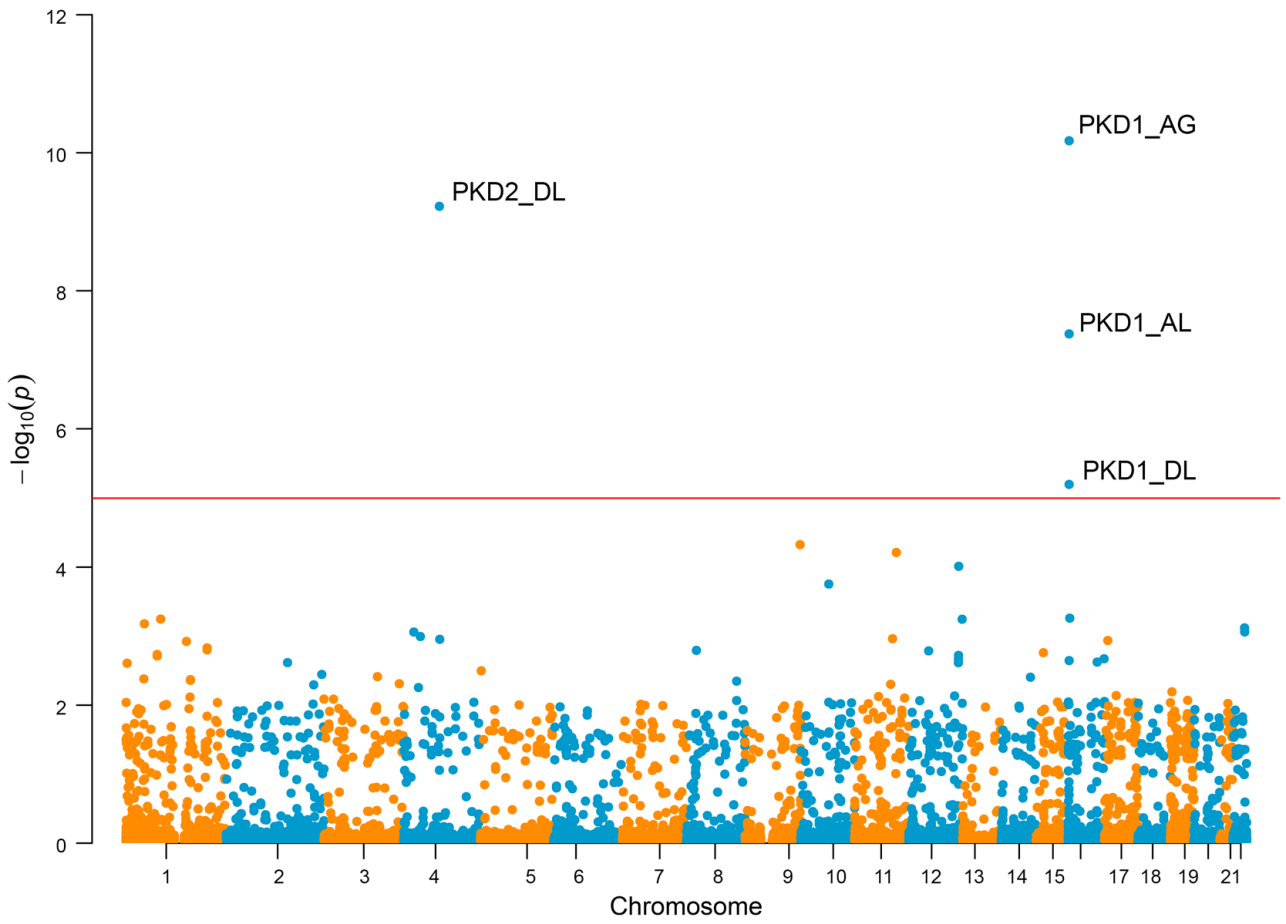
Noncoding collapsing analysis of the no-variant-detected (NVD) cohort revealed enrichment of splice-site variants in *PKD1* and *PKD2*. Gene-based Manhattan plot of SAIGE-GENE splicing analysis. Each data point is a gene representing the significance of the association with CyKD in 248 cases versus 26,096 ancestry-matched controls, made up of variants that are highly likely (SpliceAI score >0.8) to impact splicing. The horizontal line indicates the threshold for genome-wide significance. There was significant enrichment in acceptor gain (AG), acceptor loss (AL), and donor loss (DL) variants for *PKD1* (AG *P* = 6.70 × 10^–11^, AL *P* = 4.22 × 10^–8^, DL *P* = 6.32 × 10^–6^), and for DL in *PKD2* (*P* = 5.97 × 10^–10^). Generalized logistic regression modeling, as implemented using optimal sequence kernel association testing (SKAT-O) via SAIGE-GENE, was used to generate these data.

Removing the cases with qualifying *IFT140*, *COL4A3*, and monoallelic *PKHD1* variants led to no further enrichment in the no-variant-detected (NVD) cohort under the “missense+” or “LoF” gene-collapsing tests. However, in the remaining 248 cases versus 26,096 controls there was significant enrichment in acceptor gain (AG), acceptor loss (AL), and donor loss (DL) splice variants for *PKD1* (AG *P* = 6.70 × 10^–11^, OR 150.57, 95% CI 35.39–730.24; AL *P* = 4.22 × 10^–8^, OR 398.51, 95% CI 39.10–16384; DL *P* = 6.32 × 10^–6^, OR = no variants in controls) and for DL in *PKD2* (*P* = 5.97 × 10^–10^, OR = no variants in controls) (Figure 5).

There was no enrichment in the 3′- or 5′-UTR, intronic regions with a CADD score of greater than 20, or donor gain splice sites on a genome-wide basis.

### Rare variant analysis by primary variant does not reveal contribution of variants in other genes.

Using the primary variant, the CyKD cohort was divided into cases with *PKD1*- and *PKD2*-truncating and -nontruncating variants, respectively. Excluding the primary gene in each cohort did not identify significant enrichment of any additional genes.

A full list of the summary statistics that includes the variants that make up each association can be found in Supplemental Tables 5–26.

### SVs in PKD1, PKD2, and the 17q12 loci play an important role in CyKD.

**Figure 6:**
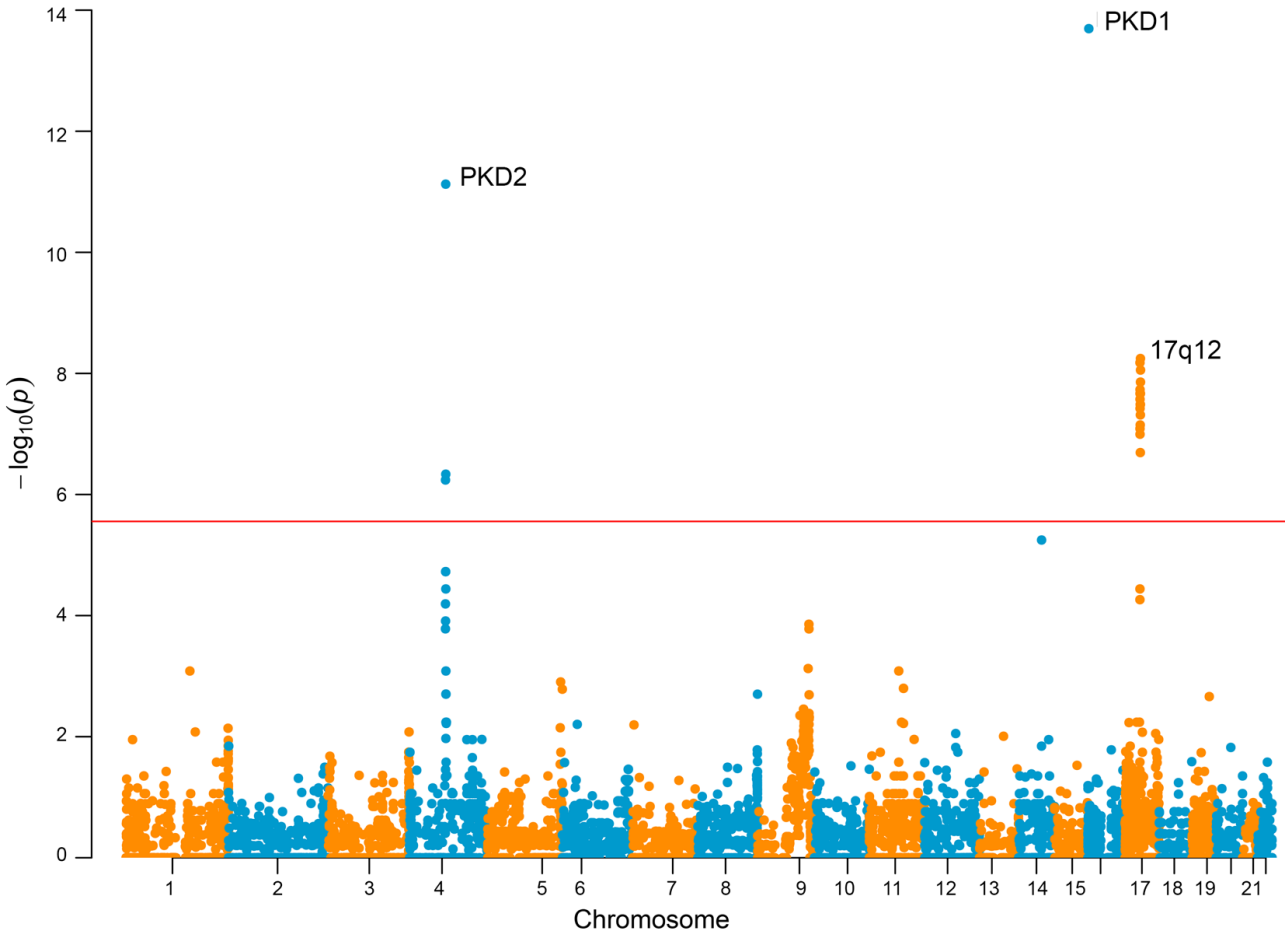
SVs in *PKD1*, *PKD2*, and the 17q12 loci play an important role in CyKD. Gene-based Manhattan plot. Each data point is a gene representing the significance of the association with CyKD in 1,209 cases and 26,096 ancestry-matched controls, made up of rare (in-house MAF < 0.01), exon-crossing SV/CNVs that have been called by MANTA/CANVAS. Common SV/CNVs (MAF > 0.1%) seen in gnomAD or the 100KGP cancer cohort were excluded. The horizontal line indicates the threshold for genome-wide significance. The significant associations were *PKD1* (*P* = 2.02 × 10^–14^), *PKD2* (*P* = 7.48 × 10^–12^), and the 17q12 locus (*P* = 8.81 × 10^–9^). A 2-sided Fisher’s exact test was utilized to compare cases and controls under a dominant inheritance model.

Exome-wide gene-based SV analysis was performed in all CyKD cases and ancestry-matched controls. Across all combined types of SV (CNVs, deletions, duplications, inversions) there was significant enrichment in *PKD1* (*P* = 2.02 × 10^–14^, OR 2.52, 95% CI 1.69–3.63), *PKD2* (*P* = 7.48 × 10^–12^, OR 3.51, 95% CI 1.74–6.37), and genes within the 17q12 locus, including *HNF1B* (*P* = 8.81 × 10^–9^, OR 7.11, 95% CI 3.41–13.66). Of note, 2 genes within proximity of *PKD2* also reached genome-wide significance: *SPARCL1* (*P* = 5.76 × 10^–7^) and *HSD17B11* (*P* = 8.69 × 10^–6^), but these were made up of large CNVs that also encompassed *PKD2* (Figure 6).

The *PKD1* signal was driven by small deletions of less than 10 kb (median size 1.14 kb, IQR 2.60) (*P* = 2.17 × 10^–22^, OR 8.11, 95% CI 4.58–13.83). For *PKD2* (*P* = 7.48 × 10^–12^, OR 13.03, 95% CI 5.02–31.87) and the 17q12 locus (*P* = 4.12 × 10^–8^, OR 8.70, 95% CI 3.72–18.80), the signal was driven by deletions of greater than 10 kb (median size in *PKD2* 405 kb, IQR 1273 kb; 17q12 1550 kb, IQR 94 kb), with no other loci reaching genome-wide significance. No genes reached genome-wide significance for duplications.

Of the 46 patients with rare exon-crossing SVs in *PKD1* or *PKD2*, 13 also harbored predicted LoF variants in *PKD1* or *PKD2*, thus leaving 33 patients with CyKD attributable to SVs in *PKD1* or *PKD2*.

Of the 11 patients with 17q12 loci CNVs in the CyKD cohort, 1 patient had a *PKD1*-nontruncating SNV and 2 had *PKD1*-truncating SNVs that met the criteria for being likely disease causing. One patient had a known *HNF1B* CNV detected by a separate diagnostic lab prior to the return of 100KGP results.

Analyzing the subgroup of patients without an identified molecular diagnosis (*n* = 248), there was significant enrichment for large (>10 kb) deletions at the 17q12 loci (*P* = 9.21 × 10^–9^, OR 24.04, 95% CI 8.00–60.71) that were detected in 7 probands.

Of the seven 17q12 patients, the median age was 13.5 years, significantly lower than the total cystic disease cohort (*P* < 0.05). None of the patients had reached KF or had HPO or HES codes pertaining to diabetes; a full breakdown of phenotypic profile can be found in Supplemental Table 27 and all summary statistics from the SV analysis can be found in Supplemental Tables 28 and 29.

### More recently described genes in CyKD are less penetrant than PKD1 and PKD2.

There was marked variation in the proportion of individuals with each gene/variant type that were documented to have CyKD, with the figures broadly comparable between 100KGP and the UK Biobank (UKBB) (Table 2).

### SeqGWAS of CyKD reveals no robust common variant associations.

A sequencing-based GWAS (seqGWAS) of 1,209 CyKD cases and 26,096 ancestry-matched controls using 10,377,275 variants with a minor allele frequency (MAF) of greater than 0.1% (Supplemental Figure 4) revealed only a single variant reaching genome-wide statistical significance on chromosome 8, chr8:92259567:A:C (*P* = 1.38 × 10^–8^, OR 0.72, MAF 0.23). There was no evidence of genomic inflation (λ = 0.99). To confirm/refute this surprising finding, we meta-analyzed this data set with those from the UKBB, Japanese Biobank (JBB), and FinnGen biobank. In the non-100KGP data sets there was evidence of association at several loci, most notably a stop gain in *PKHD1* in the FinnGen cohort (see Supplemental Figures 5 and 6 for individual study Manhattan plots), but the chr8:92259567:A:C signal was not replicated and likely to be a false positive. Overall, in the combined analysis of 2,923 CyKD cases and 900,824 controls across 6,641,351 variants there were no genome-wide significant associations (Supplemental Figure 7).

Subgroup analysis by primary disease–causing variant type did not reveal any genome-wide significant loci (see Supplemental Figures 8 and 9).

### The proportion of heritability attributable to common variants was between 3% and 9%.

Within the 100KGP CyKD cohort, the proportion of phenotypic variance (*h*^2^) explained by additive common and low-frequency variation among individuals of European ancestry was 9.0% (SEM 7.6%). Using the summary statistics from the combined FinnGen/UKBB CyKD GWAS, the estimated heritability was 3.0% (SEM 9.7%). The large SEMs reflect low power to detect heritability within this cohort.

### TTE analysis did not reveal any trans-acting genetic modifiers of severity.

Within the pre-ancestry–matched CyKD cohort, 398 of the 1,288 probands had reached KF (30.9%), with a median age of 52 years (IQR 16). TTE genetic association analysis did not reveal any genome-wide significant associations — either in the total cohort or stratified by primary gene or variant type (see Supplemental Figure 10).

## Discussion

Of the 1,209 ancestry-matched CyKD patients, 994 (82%) had a qualifying potentially pathogenic SNV or SV identified through a combination of clinical grade and unbiased research analyses of biobank-scale WGS data.

The high diagnostic yield of WGS to investigate CyKD has led to this technology being made available to patients presenting with CyKD in the UK via the National Health Service’s (NHS’s) Genomic Medicine Service (19) (though it must be noted that as yet a proportion of these variants do not necessarily meet American College of Medical Genetics and Genomics and Association for Molecular Pathology (ACMG-AMP) criteria for issuing a clinically actionable molecular diagnosis).

The data presented also clarify the underlying genetic architecture of CyKD. They point strongly to the conclusion that CyKD is extensively driven by monogenic mechanisms via rare variants of multiple different types, with a small contribution from common variants.

The arguments for this position are compelling. Firstly, this unbiased method has confirmed the importance of established and newly described genes in the pathogenesis of CyKD (*PKD1*, *PKD2*, *IFT140*, and *DNAJB11*).

Secondly, we provide robust statistical evidence that *COL4A3* is associated with CyKD. Smaller studies have hinted at this association (20), and sequencing of unexplained KF patients in an American cohort showed that a significant proportion of unexplained cystic cases were attributed to the *COL4A* family of genes (21). Using collapsing burden testing (as opposed to SKAT-O), another group has also observed enrichment of rare variants in *COL4A3* in CyKD in the 100KGP (22). In the UKBB, *COL4A4* is the most strongly associated *COL4A* gene with a CyKD phenotype (*P* = 5.85 × 10^–4^) and it is likely the play of chance, i.e., the rare variant distribution in our cohort versus UKBB, that accounts for the observation of association with *COL4A3* rather than *COL4A4* (or both genes); it is likely that a larger study would have the power to detect associations with both genes. Of note, 4 patients in the *COL4A3* cohort had liver cysts and despite no potentially pathogenic variants being found in genes associated with polycystic liver disease (*PRKCSH*, *SEC63*, and *LRP5*), we cannot completely rule out the presence of a second variant contributing to this phenotype.

Thirdly, our attempts to find common variants that might contribute to the CyKD phenotype by meta-analyzing more than 2,000 cases with nearly 1 million controls did not reveal any significant associations. In fact, in the Finnish population that has undergone significant genetic bottlenecks causing positive selection for certain recessive variants, there is an enrichment of a known pathogenic *PKHD1* variant at an allelic frequency that borders the “rare” variant mask (rs137852949, MAF in Finnish population = 7.48 × 10^–3^, MAF in non-Finnish European population = 3.24 × 10^–4^), but meets inclusion in FinnGen. This variant has been implicated as a heterozygous cause of polycystic liver disease (23) and its enrichment in our cohort as a heterozygous entity provides evidence for the role of *PKHD1* as a monoallelic cause of CyKD. Of note, 2 of the monoallelic *PKHD1* cases also exhibited hepatic fibrosis, which would be consistent with ARPKD; however, interrogation of their genomes did not reveal a plausible second variant. Potential explanations for this include that these cases had an unannotated second variant (perhaps hypomorphic rather than truly pathogenic); they had somatic mosaicism for a second pathogenic *PKHD1* allele; they had an alternative cause for hepatic fibrosis; or that monoallelic *PKHD1* variants can manifest with this phenotype. Long term follow-up or more detailed acquisition of phenotypic data from patients with monoallelic *PKHD1* variants may help to answer this in the future.

Our analysis of common variant heritability suggests that 3%–9% of CyKD may be explained by low-penetrance common variation, though our efforts to ascertain this were underpowered, resulting in large SEMs. The small magnitude of this contribution explains why individual genome-wide significant variants were not detected — the small effect size means an additional 440 cases would be required to detect a heritability signal with greater than 80% power and that substantially larger studies would be needed to detect the loci contributing to this risk. (A full power calculation can be found in Supplemental Figure 9). Our efforts to use age of KF per driving molecular diagnosis within CyKD is an attempt to try and unpick common variant contributions to disease severity and quantitatively define genetic modifiers, a key question faced by those researching CyKD, but power was not sufficient to detect any significant signals. This represents the largest systematic analysis of whether oligogenic or polygenic mechanisms are important in the etiology of CyKD and with a rapidly increasing number of CyKD patients undergoing WGS as part of routine clinical care in the UK and establishment of a National Genomic Research Library, our study sets up an analytical pathway to address this in the future with ever-increasing power.

Similar to our recent work on urinary stone disease highlighting the importance of intermediate effect rare variants as a risk factor (24), as well as work describing a low-frequency *UMOD* variant (present in 0.1% of European ancestry individuals) that confers an intermediate risk of KF (25), we show that CyKD represents another disease enriched for such variation. As shown in Table 2, the OR for a CyKD diagnosis for each gene across both the 100KGP cohort and the UKBB is highly variable. This highlights that even highly deleterious variants in some of the more recently reported CyKD genes are likely to have such a low penetrance that they may seldom exhibit Mendelian patterns of inheritance and may be perhaps regarded as intermediate risk factors for developing CyKD rather than “pathogenic” variants in the Mendelian disease sense. Communicating this information clearly to patients and their relatives is likely to be important when counseling them about the pros and cons of predictive testing or reproductive interventions for these disorders.

Finally, our findings are replicated in the UKBB with the top gene associations with CyKD being *PKD1* (*P* = 9.83 × 10^–63^), *PKD2* (*P* = 1.64 × 10^–60^), and *IFT140* (*P* = 4.52 × 10^–15^) in a European ancestry cohort of 531 patients and 239,516 controls (26). We calculate that the UKBB CyKD cohort is powered to detect genes that explain 8% or more of the total phenotypic variance, meaning genes associated with smaller effect sizes are unlikely to be identified.

The larger ORs observed for *PKD2* compared with *PKD1* is notable. We believe ascertainment bias of patients with *PKD1* variants in the 100KGP might explain this, as patients with *PKD1* variants tend to present earlier to healthcare services with hypertension and other sequelae. In an unrelated analysis of patients with severe early-onset hypertension in 100KGP, we found *PKD1* to be the most strongly associated gene on collapsing analysis, with many of the cases having been solved (27). This suggests that many CyKD-*PKD1* patients have a clinical diagnosis early in life and were therefore not entered into the 100KGP (100KGP recruitment criteria dissuaded recruitment of typical CyKD patients), meaning there are fewer *PKD1* patients (56% of those with potentially pathogenic variants) compared with the prior literature. Among 655 French patients with CyKD and a molecular diagnosis reported for clinical purposes, 80% had *PKD1* variants (28) and consistent with similar data from patients undergoing genetic testing for CyKD in a clinical setting in the UK (unpublished National Registry of Rare Kidney Diseases [RaDaR] data: 438/550 (80%) patients have *PKD1* variants). This suggests that the 100KGP and UKBB cohorts are probably depleted for people with *PKD1*-associated disease compared with people undergoing genetic testing for clinical reasons. It is also possible, however, that clinical sequencing cohorts may be susceptible to ascertainment bias, where patients with more severe or earlier-onset disease are more likely to have a molecular test. Finally, it is important to note that these ORs represent the odds of being ascertained for CyKD and should not automatically be seen as a marker for disease severity or age at KF.

Using WGS, we have also undertaken the first systematic assessment to our knowledge of the structural and noncoding variant contribution to CyKD. These contribute to unsolved cases, highlighting the power WGS has in identifying sites previously untested by traditional sequencing technologies. While splice-site variants have been implicated in individual families with unexplained CyKD (29, 30), this analysis gives quantitative statistical evidence at a population level that suggests these sites should be scrutinized in clinical analysis of *PKD1* and *PKD2*, something that is increasingly being recognized (31). Equally, utilizing methodology similar to the gnomAD SV working group (32) we find, as they did, that SVs play a larger role in the variant landscape than previously thought. While SVs in CyKD genes have been implicated in small numbers, this cohort-level analysis attributes at least 3.35% (40 of 1,209) of the cystic disease burden to SVs, a similar proportion to the recently described *IFT140* gene at a population level. These discoveries are made possible by WGS, which unlike older methods such as multiplex ligation-dependent probe amplification (MLPA) or arrays, enable accurate calling of many different types of SVs genome-wide. These findings should help inform decisions about the sensitivity of short-read WGS and other potential sequencing approaches, such as RNA sequencing or long-read DNA (33) sequencing, in a clinical setting.

In the remaining unsolved cases, we did not find any further enrichment at an SNV, gene, pathway, or SV level. Given the findings above, one explanation is that we lacked the power to detect additional monogenic signals in this group — either because they have reduced effect size or are individually extremely rare. Alternatively, it may be that a proportion of this group exhibited CyKD as a consequence of non-monogenic developmental disorders, somatic mosaicism that WGS would be unable to detect, or undocumented environmental exposures that we have not accounted for such as diet, lithium exposure (34), or smoking that are known to affect CyKD risk or phenotype and may account for some of the missing heritability. Irrespectively, this work gives an estimate of the cohort size (an additional 2,000 cases using the assumptions in the *Power* section of the Methods) needed to power future studies to discover additional monogenic causes of CyKD using unbiased genome-wide approaches. As more patients with CyKD are sequenced as part of their routine healthcare in the UK it is possible that this threshold will be passed, and further monogenic causes will be discovered using this type of methodology. Coupled with developments in analytical techniques, identification of variants across the allelic frequency and disease risk spectrum may further extend understanding of the biological basis of CyKD.

This study has several other limitations. From a phenotype perspective, we are reliant on age at KF as determined by hospital coding systems as our only marker of outcome, and were unable to access granular phenotypic and imaging data for our cohort, limiting our ability to apply several prognostic tools (11, 35) that would have aided in further stratification as well as potentially improving power by reducing the chance of misclassification of cases as controls. Secondly, while this study represents the largest WGS study in CyKD to date, we were underpowered to detect common variant signals in our seqGWAS with an OR of less than 3, and this has limited our ability to find tractable signals in all forms of GWAS as well as in the heritability analysis. The heritability estimates also relied on pre-computed taggings from the UKBB, potentially introducing biases influenced by assumptions about genetic architecture and population structure. Furthermore, while WGS allows for more accurate SV calling over traditional microarrays, this process is highly dependent on the algorithm used for calling, with the possibility of false positives. Ideally long-range sequencing and independent validation would allow for more complete SV detection. Equally, we were unable to functionally characterize the splice variants given the lack of RNA sequencing, meaning our conclusions rest on the enrichment of such variants in cases compared with controls and any clinical actionability for participants in the 100KGP would be subject to cDNA confirmation on a case-by-case basis.

In summary, this study provides the most comprehensive WGS analysis of CyKD to date, highlighting the contributions to disease risk of different types of variants across the allelic spectrum. While some of our findings (*COL4A3* and *PKHD1*) will need additional validation both functionally and statistically, these findings can be used to inform genomic sequencing and counseling strategies offered to patients. This study also provides a blueprint for the unbiased analyses of other rare diseases using WGS on a biobank scale.

## Methods

### Sex as a biological variable.

In all presented analyses sex, as determined by X and Y genotypes, was used as a covariate in all models generated.

### The 100KGP.

The 100KGP is one of the largest disease-based sequencing initiatives in the world, in which WGS data from large numbers of NHS patients with rare diseases and cancer, and their relatives, have been generated (36, 37). Key strengths of this data set with respect to the study of rare diseases are that all germline samples are processed and analyzed using a shared pipeline and that sequencing data are available for many individuals without the phenotype under study, drawn from the same population. This allows for robust control of technical artefacts, allele frequency, and variant burden in the population, in contrast with previous sequencing studies.

Recruitment to the 100KGP is via a network of 13 NHS Genomic Medicine Centres (GMCs) and includes collection of phenotype data hierarchically encoded using HPO codes (38), facilitating computerized analysis of clinical features. CyKD patients were recruited to the project if they had more than 5 cysts affecting one or both kidneys with at least one of the following features: cysts not clinically characteristic of ADPKD; onset before the age of 10; syndromic features; where a genetic diagnosis would influence management; and/or features suggestive of classical ADPKD who had not undergone prior genetic testing of *PKD1* and *PKD2*.

Participants were excluded if they suffered from KF due to identified (non-cystic) disease, if they had multicystic dysplastic kidney(s), or if they had a prior genetic diagnosis for their condition. We used the Genomics England data set (v15) (39), which contains WGS data, details of clinical phenotypes encoded using HPO terms, and structured data automatically extracted from NHS hospital records, collected for more than 90,259 cancer and rare disease patients (see *Data availability*) as well as their unaffected relatives to generate the cohorts. The study workflow and a full description of the cohort creation can be found in Supplemental Methods 1–4. After quality control, relatedness filtering and ancestry matching (Supplemental Methods 1–4 and Supplemental Figure 2), we were left with 1,209 cases and 26,096 controls for analysis.

Potentially pathogenic variants were ascertained through a combination of the clinical arm of the 100KGP and bioinformatically as detailed below to create subgroups stratified by enrichment for primary molecular cause of CyKD. We performed all SNV, gene-burden, and SV analysis in the total cohort and each molecular subgroup (except the “other genes” group). We used the same controls for each subgroup without repeating ancestry matching, as there was no evidence of genomic inflation within each subgroup and the controls (λ between 0.99 and 1.02 in all common variant analyses; see Supplemental Figure 9). We performed European-only analysis in the unexplained CyKD cohort to highlight the advantages of ancestry matching (Supplemental Methods 4 and Supplemental Tables 9 and 13).

### Single-variant seqGWAS.

Whole-genome single-variant association analysis (seqGWAS) was carried out using the R package SAIGE (40) (v0.42.1) which uses a generalized linear mixed model (GLMM) to account for population stratification. High-quality, autosomal, biallelic, LD-pruned SNVs with MAF of greater than 5% were used to generate a genetic relationship matrix and fit the null GLMM. Sex and the top 10 principal components were used as covariates (fixed effects). SNVs and indels with MAF of 0.1% or greater that passed the following quality control filters were retained: minor allele count (MAC) of 20 or greater, missingness less than 1%, Hardy-Weinberg equilibrium *P* greater than 1 × 10^–6^, and differential missingness *P* greater than 1 × 10^–5^. When case-control ratios are unbalanced, as in our study, type 1 error rates are inflated because the asymptotic assumptions of logistic regression are invalidated. SAIGE employs a saddle point approximation (41) to calibrate score test statistics and obtain more accurate *P* values than the normal distribution.

One limitation of SAIGE is that the βs estimated from score tests can be biased at low MACs, and therefore ORs for variants with MAF of less than 1% were calculated separately using allele counts in R. The R package qqman (42) was used to create Manhattan and Q-Q (quantile-quantile) plots. The genomic inflation factor (λ), calculated based on the 50th percentile, was between 0.99 and 1.02 in all analyses, indicating no significant population stratification.

### Meta-analysis of GWAS data.

A meta-analysis of CyKD GWAS using summary statistics from our analysis, a combined UKBB/JBB analysis of 220 phenotypes, including PKD (19,093,042 variants) and FinnGen (version 8) analysis of CyKD (43) (19,441,692 variants) was performed using METAL (version 2011-03-025) (44). The summary statistics from the UKBB/JBB analyses were lifted over from build 37 to 38 using the UCSC liftover tool (45). A full breakdown of the biobank phenotypes can be found in Supplemental Methods 5. Between the 3 data sets, 8,217,458 variants were shared with matching alleles. Meta-analysis was performed weighting the effect size estimates using the inverse of the SEMs. Variants showing heterogeneity of effect between the 2 data sets (*P* < 1 × 10^−5^) and those in which the MAFs/maximum allele frequencies differed by more than 0.05 were excluded, leaving 6,641,352 variants across 2,923 cases and 900,824 controls. The genomic inflation factor (λ), calculated based on the 50th percentile, was 1.01, indicating no significant population stratification.

### Single-variant seqGWAS TTE analysis.

Genetic analysis of TTE phenotypes (GATE) (46) was used to conduct a TTE analysis, utilizing the Cox proportional hazard model that accounts for heavily censored phenotypes and low-frequency variants. The 100KGP project participants consented to give access to their HES, which is a database containing details of all admission, emergency attendances, and outpatient appointments at NHS hospitals in England. The database was searched for codes (full list of codes used in Supplemental Table 32) that would highlight whether a patient had reached KF as well as codes pertaining to their stage of CKD from stage 1 to 5 (Supplemental Table 1). The age of KF, as determined by the earliest occurrence of a clinical code for KF, was used as the end point in the TTE analysis and those who were yet to reach KF were censored. The same genomic and phenotype data as per the single-variant seqGWAS was used to conduct the TTE GWAS.

### Heritability estimation using common variants.

Narrow sense heritability (*h*^2^), the contribution of phenotypic variation from additive genetic factors, was estimated using 2 methods: GCTA-LDMS (47) and LDAK-SumHer (48). GCTA-LDMS was applied to the 100KGP WGS data using a European subset of the total ancestry-matched CyKD cohort (full details in Supplemental Methods 6).

Summary statistics from the CyKD analysis of the combined European ancestry FinnGen biobank and UKBB (780 FinnGen cases and 424 UKBB cases, with 375,708 FinnGen controls and 417,905 UKBB controls) were used with LDAK-SumHer to calculate heritability under the BLD-LDAK model using the pre-computed taggings (a record of the relative expected heritability tagged by various predictors), calculated from the UKBB.

The observed heritability was then liability adjusted to account for the population prevalence of CyKD relative to its representation in the 100KGP (49). In this analysis, a CyKD prevalence of 0.001 was used to transform the observed heritability to a liability threshold model.

### Rare variant collapsing analysis.

Prior to collapsing analysis, variants were filtered based on different criteria-called masks. The masks used for this analysis were a rare, damaging missense mark (“missense+”), a high confidence LoF mask (“LoF”), an intronic mask (“intronic”), a splice site mask, a 3′-UTR mask, and a 5′-UTR mask. For the total CyKD cohort and the unsolved NVD cohorts, we also ran a rare exome variant ensemble learner–derived (REVEL-derived) (50) mask to investigate missense signals in more detail. Full details of the mask parameters and quality control can be found in Supplemental Methods 7.

We applied the “missense+” and “LoF” masks to the total cohort and then removed cases that had qualifying variants in statistically significant genes until we had a cohort of patients with NVD. To this cohort we applied all the masks listed above, looking for previously undetected gene signals. Association testing was performed using the Scalable and Accurate Implementation of Generalized mixed model (SAIGE-GENE) (v0.42.1) (51) to ascertain whether rare coding variation was enriched in cases on a per-gene basis exome wide. Sex and the top 10 principal components were included as fixed effects when fitting the null model. Full details about the use of SAIGE can be found in Supplemental Methods 8.

### Subgroup analysis stratified by primary variant and depleting analysis.

Patients who had their phenotype “solved” by the clinical multidisciplinary team (MDT) had a report issued with the details of the molecular diagnosis. Depending on the diagnosis, these patients were placed into different cohorts: *PKD1*-truncating (*PKD1*-T), PKD1–non-truncating (*PKD1*-NT), *PKD2*-truncating (*PKD2*-T), *PKD2*–non-truncating (*PKD2*-NT), “other gene” (encompassing other genes in the panel), and NVD. In the patients with NVD, we bioinformatically reanalyzed them, looking for variants that met the “missense+” or “LoF” mask (detailed below), in the approved CyKD panel of genes in PanelApp (52) and placing them in the relevant cohort. The filtering was performed using bcftools and filter-VEP. For each subsequent round of analysis (across SNV and SVs), if a gene or SV was found to be significantly enriched in cases, we identified the cases that contained qualifying variants and removed them from the NVD cohort and reanalyzed the cohort, eventually leaving 184 cases with no clear genetic cause of disease identified.

We performed all SNV, gene-burden, and SV analysis in each molecular subgroup (bar the “other genes” group). We used the same controls for each subgroup without repeating ancestry matching as there was no evidence of genomic inflation within each subgroup and the controls (λ between 0.99 and 1.02 in all common variant analyses; see Supplemental Figure 9).

### Pathway analysis.

For the cohort of patients that had no molecular diagnosis, the summary statistics from their rare variant SKAT-O analysis with SAIGE was analyzed using the gene set analysis association using sparse signals method (GAUSS) (53) with default settings. The summary statistics were analyzed using the canonical (3,759 pathways) and hallmark (50 pathways) curated gene set pathways from the gene set enrichment analysis (GSEA) group (54). The results of these analyses can be found in Supplemental Tables 30 and 31.

### Exome-wide SV analysis.

SVs were called from WGS using the Genomics England pipeline that incorporates CANVAS (55) to detect copy number (>10 kb) and MANTA (56) to identify SVs greater than 50 bp. CANVAS uses read depth to assign CNV losses and gains. MANTA uses both discordant read-pair and split-read data to identify SV regions. While MANTA can detect deletions and tandem duplications of less than 10 kb, inversions, and interchromosomal translocations, it cannot reliably identify dispersed duplications, small inversions (<200 bp), fully assembled large insertions (>2 × 150 bp), or breakends where repeat lengths approach the read size (150 bp). Very few insertions were identified in this cohort using MANTA and in view of this they were excluded from downstream analysis. In addition, variants classified as translocations, single breakends, or complex SVs, which are more difficult to accurately resolve, were filtered out.

The following quality control filters were applied to the variants: CNV length greater than 10 kb and *Q*-score of Q10 or greater, indicating 90% confidence there is a variant present; a quality score of 20 or greater, indicating 99% confidence that there is a variant at the site; genotype quality (GQ) of 15 or greater, indicating 95% confidence that the genotype assigned to a sample is correct; and fraction of reads with mapping quality zero around breakends (MaxMQ0Frac) less than 0.4, which indicates the proportion of uniquely mapped reads around either breakend. Variants without paired read support, inconsistent ploidy, or depth greater than 3 times the mean chromosome depth near one or both breakends were excluded.

For each sample, BEDTools (57) was used to extract SVs that intersected at least one exon by a minimum of 1 bp. Variants were then separated by type into CNV, deletion (DEL), duplication (DUP), and inversion (INV) sets before being filtered using BEDTools to remove common SVs of the same type. SVs were removed if they had a minimum 70% reciprocal overlap with the gnomAD SVs (32) with allele frequency greater than 1% and/or a data set of common (allele frequency > 0.1%) SVs generated from 12,243 cancer patients recruited to 100KGP. SVs were then merged using SURVIVOR (58), allowing a maximum distance of 300 bp between pairwise breakpoints and allele frequencies calculated using BCFtools (59).

After removal of overlapping common variants, a custom Perl script (Helen Griffin, Newcastle University, Newcastle upon Tyne, UK) was used to calculate allele frequencies for each type of SV across the combined case-control cohort using bins of 10 kb across the entire genome. SVs with an allele frequency of less than 0.1% were retained for further analysis.

Exome-wide gene-based burden testing was carried out using custom R scripts stratified by SV type. SVs were aggregated across 19,005 autosomal protein-coding genes.

### Power.

PAGEANT (60) is a power calculation tool for rare variant collapsing tests that uses the underlying distribution of gene size and MAF of variants from the ExAC data set (61). Under the assumption that 80% of variants collapsed per gene in the SAIGE-GENE analysis were causal, we calculated that we had greater than 80% power to detect a gene signal that accounted for greater than 4% of the variance of the phenotype with an exome-wide significance threshold of *P* equal to 2.5 × 10^–6^ in the total case control cohort.

For SNV analysis, power was calculated using the R package genpwr (62) under an additive model using the conventional genome-wide significance threshold of *P* less than 5 × 10^–8^. With this sample size at an allele frequency of 1%, single variant association testing was sufficiently powered (>80%) to detect alleles with an OR of greater than 3. Supplemental Figure 11 shows the results of the power calculations for the CyKD GWAS in greater detail.

For heritability analysis, we used the GCTA-GREML power calculator (63), which revealed we had a 54% chance of detecting 5% heritability within the 100KGP cohort. This is likely to be even lower in the summary statistics methods applied to the combined UKBB/FinnGen meta-analysis.

### Statistics.

For SNV association analyses (including time to event analyses), a significance threshold of *P* < 5 × 10^-8^ was applied for genome-wide statistical significance. For gene-based rare variant analyses, a Bonferroni-corrected significance threshold of *P* less than 2.58 × 10^–6^ (0.05/19,364 genes) was used. Binary ORs and 95% CIs were calculated for exome-wide significance genes by extracting the number of cases and controls carrying qualifying variants per gene in the collapsing analysis and applying a 2-sided Fisher’s test.

For pathway analysis, a 2-sided Fisher’s exact test was utilized to compare cases to controls with a Bonferroni-corrected significance threshold of *P* less than 0.05/(*n* = pathways tested).

For SV analysis, a 2-sided Fisher’s exact test was utilized to compare cases and controls under a dominant inheritance model, with a Bonferroni-corrected significance threshold of *P* less than 2.5 × 10^–6^, although with the knowledge that this is likely to be too stringent given that the tests are not truly independent (1 SV can affect multiple genes).

When comparing demographic and phenotypic characteristics between cases and controls, Student’s *t* tests and ANOVA were applied as appropriate. All *t* tests were 2-tailed unless otherwise specified. Two-way ANOVA was used when examining interactions between multiple factors.

The statistical significance for all tests was determined using a *P*-value threshold of 0.05, unless specified otherwise for specific analyses. All statistical analyses were performed in R.

### Study approval.

Ethical approval for the 100KGP was granted by the Research Ethics Committee for East of England – Cambridge South (REC Ref: 14/EE/1112). Participants provided written informed consent for the use of their genetic and clinical data.

### Data availability.

All collapsing gene and pathway analyses can be found in the Supplemental Tables. All seqGWAS summary statistics can be found at https://zenodo.org/records/10613736 Details of the aggregated data set used for the analysis can be found at https://re-docs.genomicsengland.co.uk/aggv2/ Genomic and phenotypic data from participants recruited to the 100KGP can be accessed by application to Genomics England Ltd (https://www.genomicsengland.co.uk/about-gecip/joining-research-community/).

### Code availability.

Code used for the analyses in this paper can be found at https://github.com/oalavijeh/cykd_paper Details of the ancestry derivation methods used by Genomics England can be found at https://re-docs.genomicsengland.co.uk/gen_sim/ Details of the rare variant workflow can be found at https://re-docs.genomicsengland.co.uk/avt/ Details of the common variant GWAS workflow can be found at https://re-docs.genomicsengland.co.uk/gwas/

## Author contributions

DPG conceived the study. OSA conducted the analyses and wrote the manuscript, with extensive input from MMYC, DPG and APL. GTD aided with analyses. CDV provided scripting support. AS, AK, and ATH provided bioinformatic assistance from the Genomics England side. HCS, DB, RNS, APL, and DPG provided tutelage and guidance throughout.

## Supplementary Material

Supplemental data

ICMJE disclosure forms

Supporting data values

## Figures and Tables

**Table 2:**
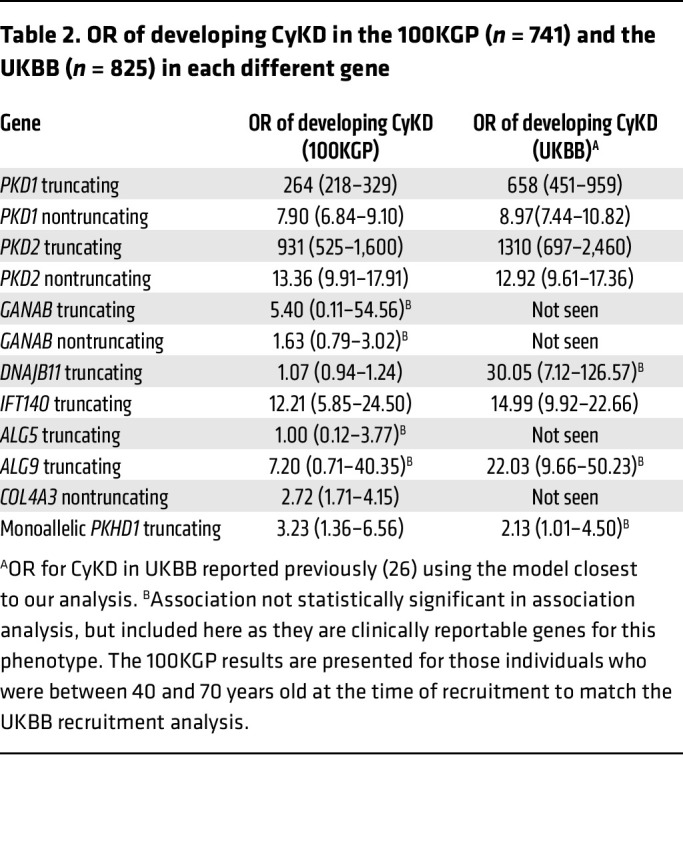
OR of developing CyKD in the 100KGP (*n* = 741) and the UKBB (*n* = 825) in each different gene

**Table 1:**
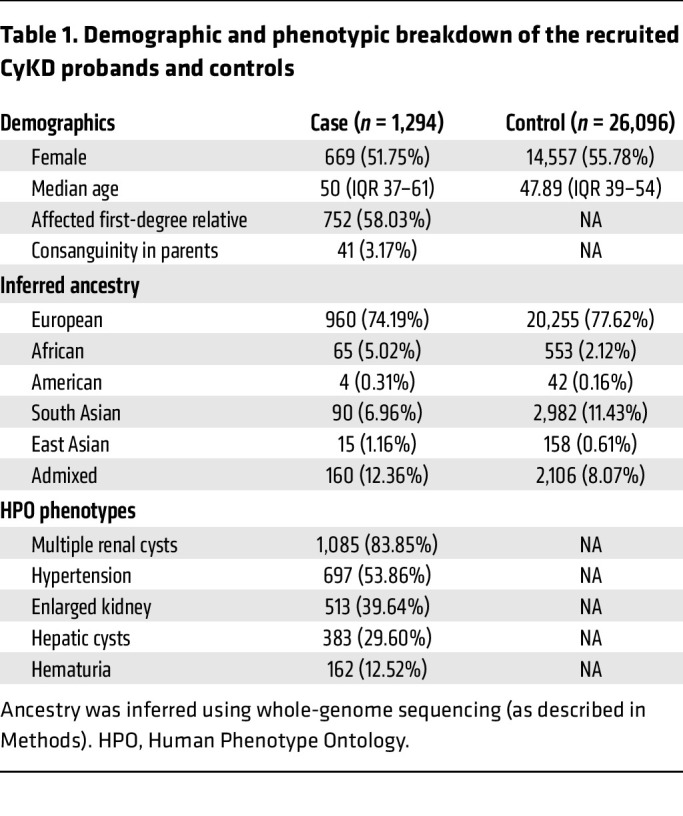
Demographic and phenotypic breakdown of the recruited CyKD probands and controls

## Version 1 08/27/2024

In-Press Preview

## Version 2 10/01/2024

Electronic publication
