# Supplementary material for: Quantifying variant contributions in cystic kidney disease using national-scale whole-genome sequencing
Source: J Clin Invest. 2024 Aug 27;134(19):e181467. doi: 10.1172/JCI181467 (PMC11444187; doi:10.1172/JCI181467)
Supplement: Supplemental data [file jci-134-181467-s124.pdf]

Quantifying variant contributions in cystic kidney disease using national-scale whole genome sequencing. - Supplementary appendix and figures

Contents

Supplementary Methods

Supplementary Methods 1: Cohort creation and rational

Supplementary Methods 2: Genome sequencing, variant calling format file annotation and variant-level quality control

Supplementary Methods 3: Relatedness estimation and principal components analysis

Supplementary Methods 4: Ancestry inference and ancestry-matching of cases and controls

Supplementary Methods 5: Other biobanks used for meta-analysis.

Supplementary Methods 6: Heritability using common variants.

Supplementary Methods 7: Rare variant selection for collapsing analysis.

Supplementary Methods 8: Background to using SAIGE-GENE.

Figures

Supplementary Figure S1 – Coverage of key genes

Supplementary Figure S2 - Ancestry matching.

Supplementary Figure S3 - Rare variant analysis of the no variant detected cohort.

Supplementary Figure S4 – 100KGP CyKD GWAS

Supplementary Figure S5 – Finngen CyKD GWAS

Supplementary Figure S6 – UKBB/JBB CyKD GWAS

Supplementary Figure S7 - Metanalysis of CyKD GWAS reveals no replicable common variant associations.

Supplementary Figure S8 - Manhattan plots of GWAS by primary driving variant

Supplementary Figure S9 - QQ plots of primary variant GWAS

Supplementary Figure S10 - Manhattan plots of time-to-event GWAS in the total CyKD and primary driving variant cohorts.

Supplementary Figure S11 – Power calculations for the CyKD GWAS

References

Supplementary Methods

Supplementary Methods 1: Cohort creation and rational

Cases were included if they were recruited under the primary diagnosis of “cystic kidney disease”. All cases recruited had been assessed in the clinical interpretation arm of the 100KGP. This involved ascertainment of variants in an expert curated panel of 28 cystic genes within PanelApp (https://nhsgms-panelapp.genomicsengland.co.uk/panels/283/v4.0) with multi-disciplinary review and application of American College of Molecular Genetics (ACMG) criteria to determine pathogenicity (1). The control cohort consisted of 27,660 unaffected relatives of non-renal rare disease participants, excluding those with HPO terms and/or hospital episode statistics (HES) data consistent with secondary causes of kidney disease or kidney failure (see supplementary table 32 for a list of codes). By utilizing a case-control cohort sequenced on the same platform, we aimed to minimize confounding by technical artefacts.

Whole genome sequencing was performed by Genomics England and described in more detail Supplementary Methods 2.

Cohort rationale

Given the small number of recruited cases, we chose to jointly analyze individuals from diverse ancestral backgrounds, thereby preserving sample size and boosting power, to do this we employed ancestry-matching of cases and controls using weighted principal components (2). To mitigate confounding due to population structure whilst using this mixed ancestry approach we utilized a generalized logistic mixed model to account for cryptic relatedness between individuals; further details can be found in Supplementary Methods 3.

Supplementary Methods 2: Genome sequencing, variant calling format file annotation and variant-level quality control

Genome sequencing was performed with the use of the TruSeq DNA polymerase-chain-reaction (PCR)–free sample preparation kit (Illumina) on a HiSeq 2500 sequencer, which generates a mean depth of 32× (range, 27 to 54) and a depth greater than 15× for at least 95% of the reference human genome. Whole-genome sequencing reads were aligned to the Genome Reference Consortium human genome build 38 (GRCh38) with the use of Isaac Genome Alignment Software. Family-based variant calling of single-nucleotide variants (SNVs) and insertion or deletions (indels) for chromosomes 1 to 22, the X chromosome, and the mitochondrial genome (mean coverage, 2814×; range, 142 to 16,581) was performed with the use of the Platypus variant caller (3).

All genomic files were aligned to the GRCh38 reference human genome (4). Genomic variant call format files (gVCFs) were aggregated using gvcfgenotyper (Illumina, version: 2019.02.26) with variants normalized and multi-allelic variants decomposed using vt (5) (version 0.57721). Variants were retained if they passed the following filters: missingness ≤ 5%, median depth ≥ 10, median GQ ≥ 15, percentage of heterozygous calls not showing significant allele imbalance for reads supporting the reference and alternate alleles (ABratio) ≥ 25%, percentage of complete sites (completeGTRatio) ≥ 50% and P value for deviations from Hardy-Weinberg equilibrium (HWE) in unrelated samples of inferred European ancestry ≥ 1×10-5. Male and female subsets were analyzed separately for sex chromosome quality control. Per-variant minor allele count (MAC) was calculated across the case-control cohort, MAC is defined as the number of minor alleles counted for each marker. Annotation was performed using Variant Effect Predictor (6) (VEP, version 98.2) including CADD (7) (version 1.5), and allele frequencies from publicly available databases including gnomAD (8) (version 3) and TOPMed (9) (Freeze 5). Variants were filtered using bcftools (10) (version 1.11).

Supplementary Methods 3: Relatedness estimation and principal components analysis

A set of 127,747 high quality autosomal LD-pruned biallelic single nucleotide variants (SNVs) with a minor allele frequency (MAF) > 1% was generated using PLINK (11) (v1.9), MAF is defined as the frequency at which the second most common allele occurs in a given population. SNVs were included if they met all the following criteria: missingness < 1%, median GQ ≥ 30, median depth ≥ 30, AB Ratio ≥ 0.9, completeness ≥ 0.9. Ambiguous SNVs (AC or GT) and those in a region of long-range high LD (Linkage Disequilibrium) were excluded. LD pruning was carried out using an r^2 threshold of 0.1 and window

of 500kb. SNVs out of HWE in any of the AFR, EAS, EUR or SAS 1000 Genomes populations were removed (pHWE < 1 ×10-5). Using this variant set, a pairwise kinship matrix was generated using the PLINK2 implementation of the KING-Robust algorithm (12) and a subset of unrelated samples was ascertained using a kinship coefficient threshold of 0.0884 (2nd degree relationships). Ten principal components were generated using PLINK2 for ancestry-matching and as covariates in the association analyses.

Supplementary Methods 4: Ancestry inference and ancestry-matching of cases and controls

Inferred ancestry was calculated by Genomics England centrally using 70,8225 high-quality (HQ) SNPs. For each individual their genetic similarity to worldwide populations in the UK Biobank were assigned using the ukbb-assigner tool (linked in the code availability section). HQ sites were intersected with 5,816,590 loci aligned to GRCh37 detailed (converted to GRCh38 coordinates using UCSC Liftover when necessary) resulting in 55,706 sites used for inference.

The big_prodMat() function from the bigsnpr R package (13) was used to project participant genotypes and reference group allele frequencies at these 55,706 sites onto the top 16 linkage-disequilibrium scaled principal components (PC1-PC16) calculated using a selection of individuals from the UK Biobank and the 1,000 Genomes Project as calculated in Prive et al 2022 (14).
Squared Euclidean distance between each participant and 21 curated reference groups on PC space were converted into approximate FST (14). Obtaining approximate FST to reference groups after SNP intersection and projection takes approximately 0.5 seconds. Coefficients of proportional genetic similarity to each of these 21 reference groups were calculated using an extension of the Summix method also described in Prive et al 2022. These projected ancestries were then collapsed into super-populations using simple grouping code in R.

Given the mixed-ancestry composition of the cohort we employed a case-control ancestry-matching algorithm to optimize genomic similarity and minimize the effects of population structure as previously described (2) with each case having to match a minimum of two controls to be included in the final cohort. 1209 cases and 26096 controls remained for analysis (Supplementary Figure S2).

Supplementary Methods 5: Other biobanks used for meta-analysis.

All other biobanking studies used imputed datasets and various genotyping platforms which are detailed in their respective publications (15,16). The Japanese Biobank (JBB) took 510 cases and 178,216 controls recruited from 12 medical institutions in Japan of predominant Japanese ancestry with a diagnostic tag of "polycystic kidney disease". GWAS was conducted in this cohort using SAIGE (v.0.37) using the top 20 principal components and age. The UK Biobank (UKBB) analysis consisted of 424 cases and 355,431 controls matched to the JBB cohort via phecodes, the association study was conducted using SAIGE and the same principal components. The FinnGen analysis consisted of 780 cases and 341,081 controls, cases were recruited if they had the ICD-10 code for cystic kidney disease (Q61) with the association analysis being conducted with REGENIE (17).

Supplementary Methods 6: Heritability using common variants.

GCTA-LDMS was applied to the 100KGP WGS data using a European subset of the total ancestry-matched CyKD cohort. Variants with MAF ≥ 0.1% were included. Using the GREML-LDMS approach variants were stratified into seven different bins based on MAF (0.001-0.01, 0.01-0.05, 0.05-0.1, 0.1-0.2, 0.2-0.3, 0.3- 0.4, 0.4-0.5) and for each bin of variants, SNP-based LD scores were calculated over a 200kb region (with 100kb overlap between two adjacent segments). For a given bin of variants defined by MAF, variants were further stratified into quartiles using LD scores. For each of the 28 bins subset by MAF and LD, GCTA was used to produce a GRM from the raw genotype files. The REML (restricted maximum likelihood) function was then used to conduct a GREML-LDMS analysis using the 28 GRMs, including the top four principal components as covariates. GREML has not been validated for mixed ancestry cohorts, so an estimation was made using a European subset of the CyKD cohort as defined by principal component analysis (903 cases and 20255 controls).

Summary statistics from the cystic kidney disease analysis of the combined European ancestry Finngen and UK biobank (865 FinnGen cases, 505 UKBB cases, 375708 FinnGen controls and 417905 UKBB controls) were used with LDAK-SumHer to calculate heritability under the BLD-LDAK model using the pre-computed taggings, calculated from the UK Biobank.

The observed heritability was then liability adjusted to account for the population prevalence of CyKD relative to its representation in the 100KGP (18). In this analysis a CyKD prevalence of 0.001 was used to transform the observed heritability to a liability threshold model.

Supplementary Methods 7: Rare variant selection for collapsing analysis.

Single variant association testing is underpowered when variants are rare and a collapsing approach which aggregates variants by gene or genomic region can be adopted to boost power. Whilst the variants are aggregated by gene, they are also filtered to help boost power. Each set of filters are applied as a "mask" which instructs the workflow to collapse variants as per a set of parameters. All cited tools were applied via the Ensemble variant effect predictor (VEP) command line tool [version 99] (6). The mask used for this analysis were a rare, damaging missense mark ("missense+"), a high confidence loss of function mask ("LoF"), an intronic mask ("intronic"), a splice site mask, a 3-prime untranslated region mask (3'-UTR) and a 5-primte UTR mask (5'-UTR). For the total CyKD cohort and the unsolved NVD cohorts we created a rare exome variant ensemble learner (REVEL) (19) derived mask to perform sensitivity analysis of the missense+ mask.

For the missense+ mask we extracted coding SNVs and indels with MAF < 0.01% in gnomAD annotated with one of the following: missense, in-frame insertion, in-frame deletion, start loss, stop gain, frameshift, splice donor, splice acceptor for each gene and further filtered them by CADD (7) (v1.5) score using a threshold of ≥20 corresponding to the top 1% of all predicted deleterious variants in the genome. Variants meeting the following quality control filters were retained: MAC ≤ 20, median site-wide sequencing depth in non-missing samples > 20 and median GQ ≥ 30. Sample-level QC metrics for each site were set to minimum depth per sample of 10, minimum GQ per sample of 20 and ABratio P value > 0.001.

Variants with significantly different missingness between cases and controls ($P<10^{-5}$) or >5% missingness overall were excluded. For the dedicated loss-of-function (LoF) analysis, variants were selected that were deemed to be of "high confidence" (HC) by the loss-of-function transcript effect estimator (LOFTEE) (8). These assessed variants are stop-gained,

splice site disrupting or frameshifts and collapsed on a per-gene basis. The same quality controls were applied as above. For the splice site mask, variants were collapsed if their SpliceAI scores were greater than 0.8 (on a scale of 0-1) indicating variants that were highly likely to affect splicing. These were divided by their predicted effect on splicing (donor loss, donor gain, acceptor loss and acceptor gain). The intronic mask was defined by variants labelled as "intronic" by VEP with a CADD score greater than 20 and with a MAF<0.01% in gnomAD. The 5' and 3' UTR masks were defined by variants with a MAF<0.01% in gnomAD, a CADD score greater than 10 and found within their respective UTR region as labelled by VEP.

REVEL is a computational tool designed to predict the pathogenicity of missense variants in human genes. It is an ensemble method that integrates various individual predictors, including scores from tools such as SIFT, PolyPhen-2 and MutationTaster. REVEL's output is a single score that reflects the likelihood of a missense variant being disease-causing. By leveraging a large dataset of known pathogenic and benign variants, REVEL is trained to distinguish between these classes effectively. Its scores range from 0 to 1, with higher scores indicating a higher probability of pathogenicity. For the REVEL missense analysis, we applied REVEL scores via VEP using a threshold of 0.8 (which seemed most analogous to a CADD_PHRED score of 20 or greater).

Supplementary Methods 8: Background to using SAIGE-GENE.

SAIGE-GENE (20) uses a generalized mixed-model to correct for population stratification and cryptic relatedness as well as a saddle point approximation and efficient resampling adjustment to account for the inflated type 1 error rates seen with unbalanced case-control ratios. It combines single-variant score statistics and their covariance estimate to perform SKAT-O (21) gene-based association testing, upweighting rarer variants using the beta (1,25) weights option. SKAT-O is a combination of a traditional burden and variance-component test and provides robust power when the underlying genetic architecture is unknown.

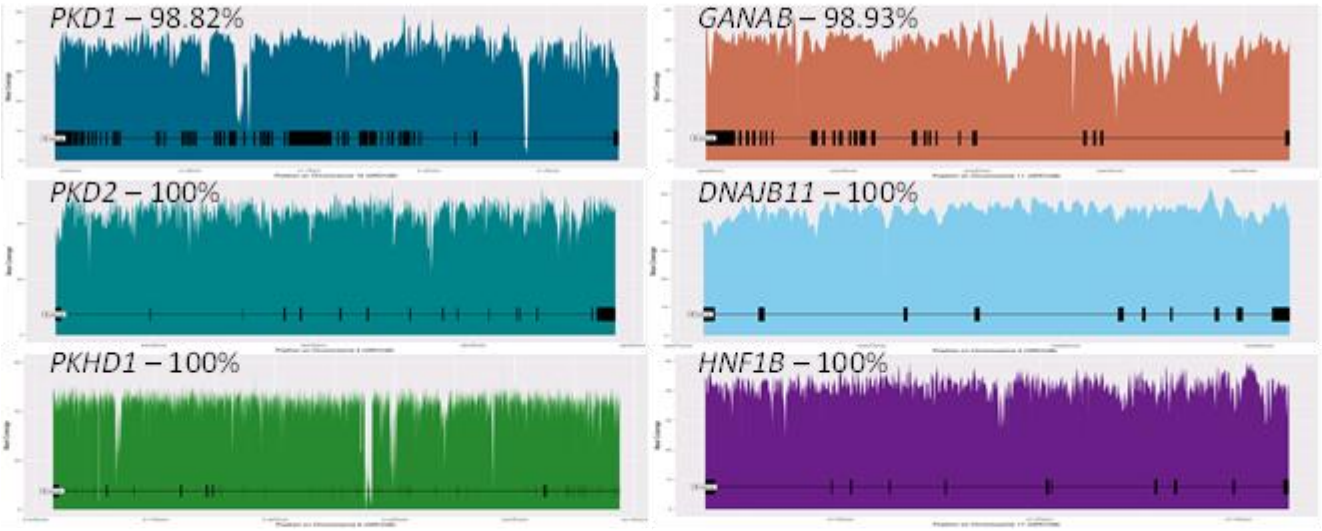

Supplementary Figure S1 – Coverage of key genes

Coverage of genes associated with cystic kidney disease, showing percentage of coding bases sequenced by mean >30 (dotted lines) uniquely mapped high-quality reads among 1209 participants with cystic kidney disease in the 100,000 Genomes Project.

2a

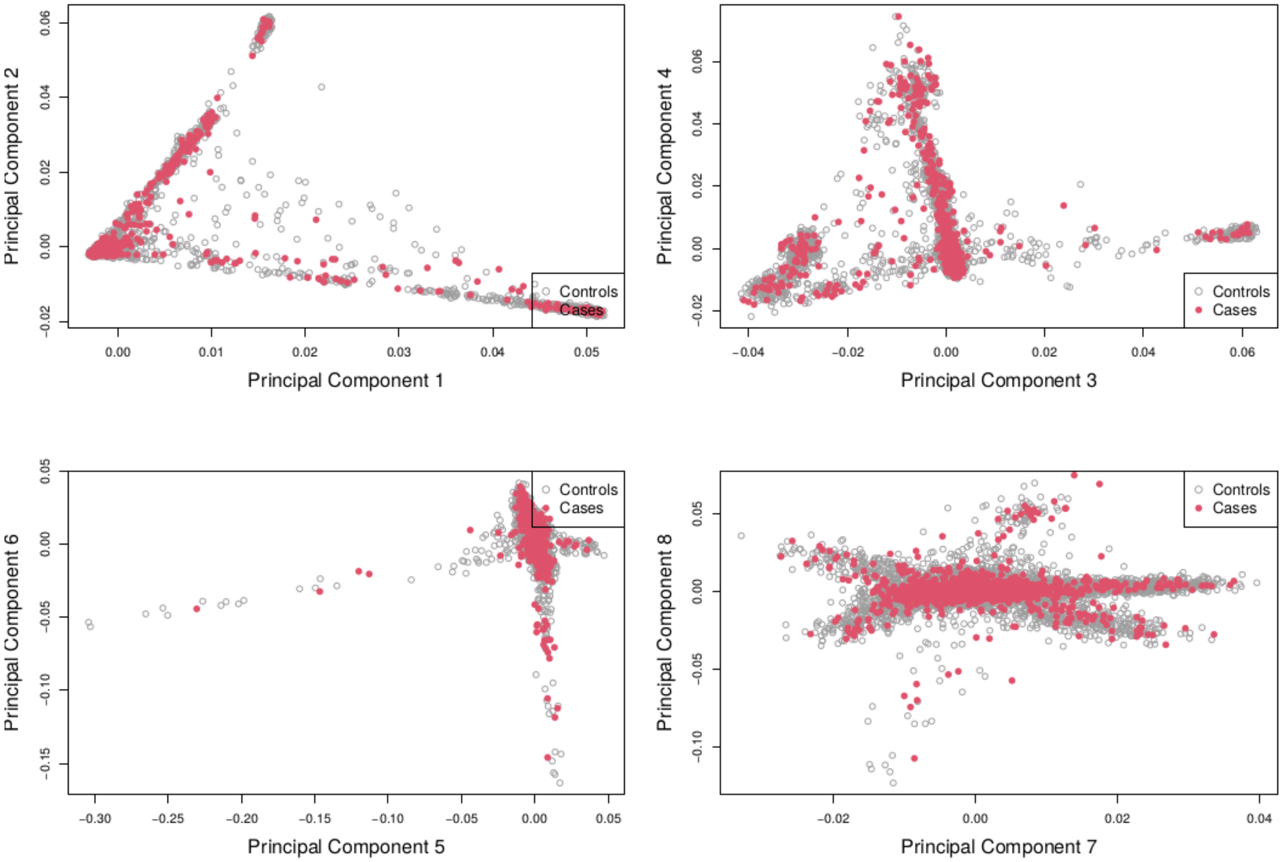

2b

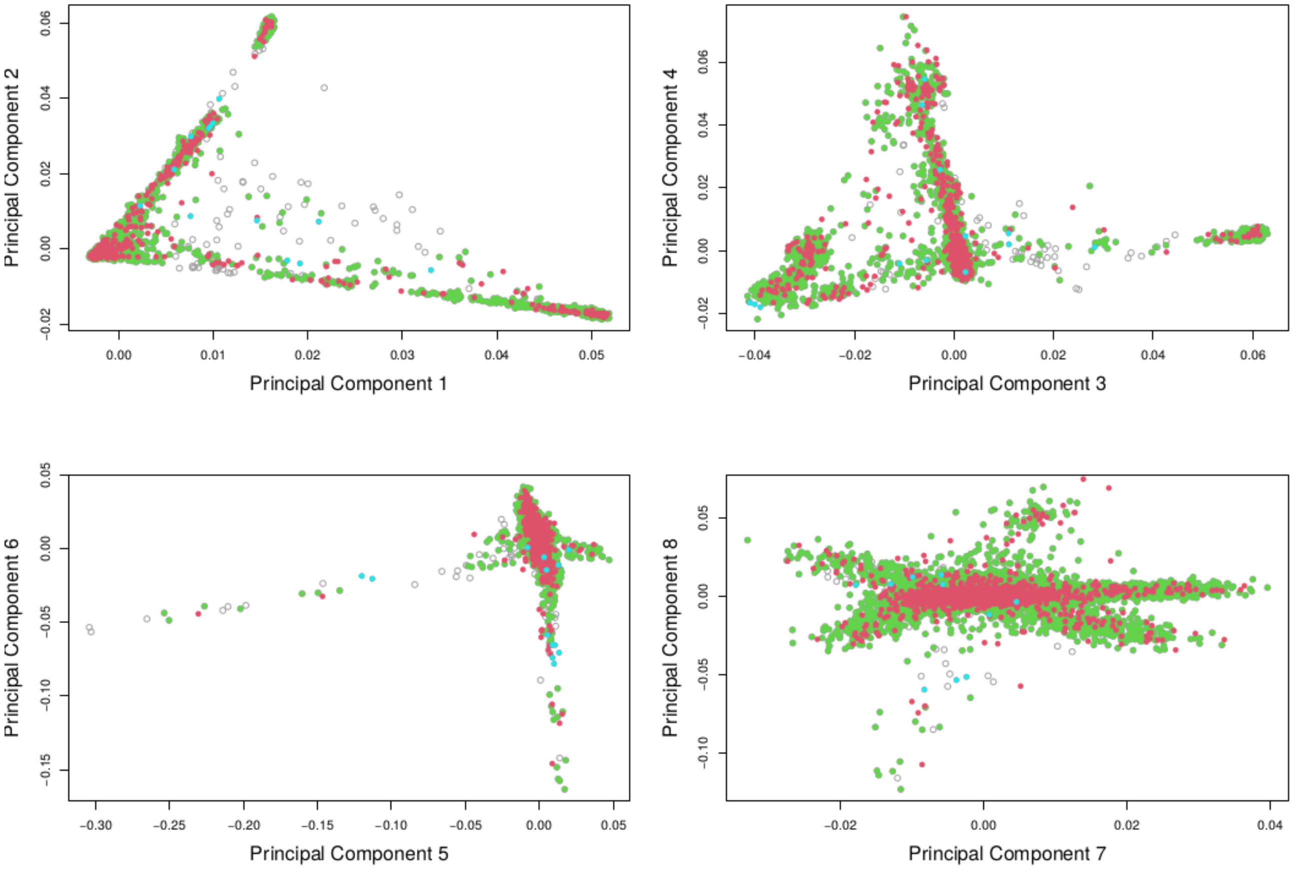

Supplementary Figure S2 - Ancestry matching.

1a. Principal component analysis showing the first eight principal components for matched cases (red) and controls (white). 1b. Principal component analysis showing the first eight principal components for matched cases (red) and controls (green) and unmatched controls (grey). This highlights that cases are taken from multiple different ancestries with the appropriate matched controls.

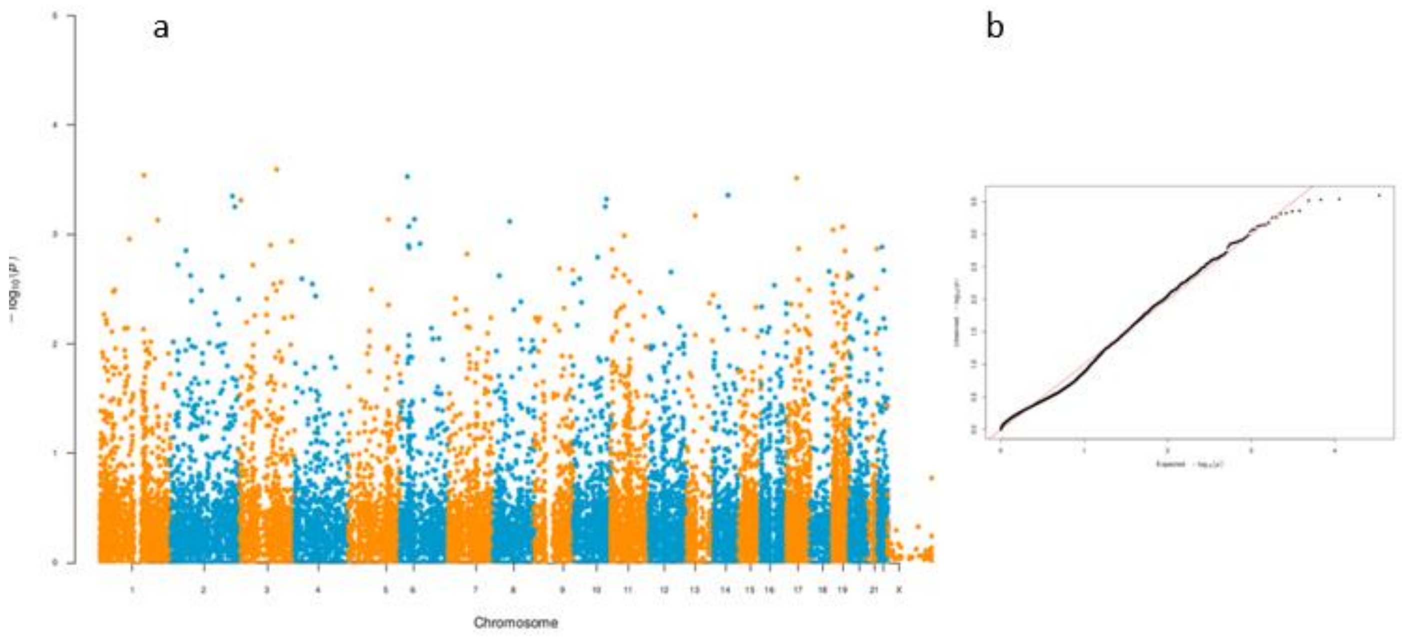

Supplementary Figure S3 Rare variant analysis of the no variant detected cohort.

2a Rare variant analysis of the no variant detected cohort depleted for COL4A3 and IFT140 cases including 266 cases and 26096 controls under the "missense+" mask showing no enrichment of genes. 2b - quantile-quantile plot of the rare variant burden analysis of 266 cases and 26096 controls under the "missense+" mask.

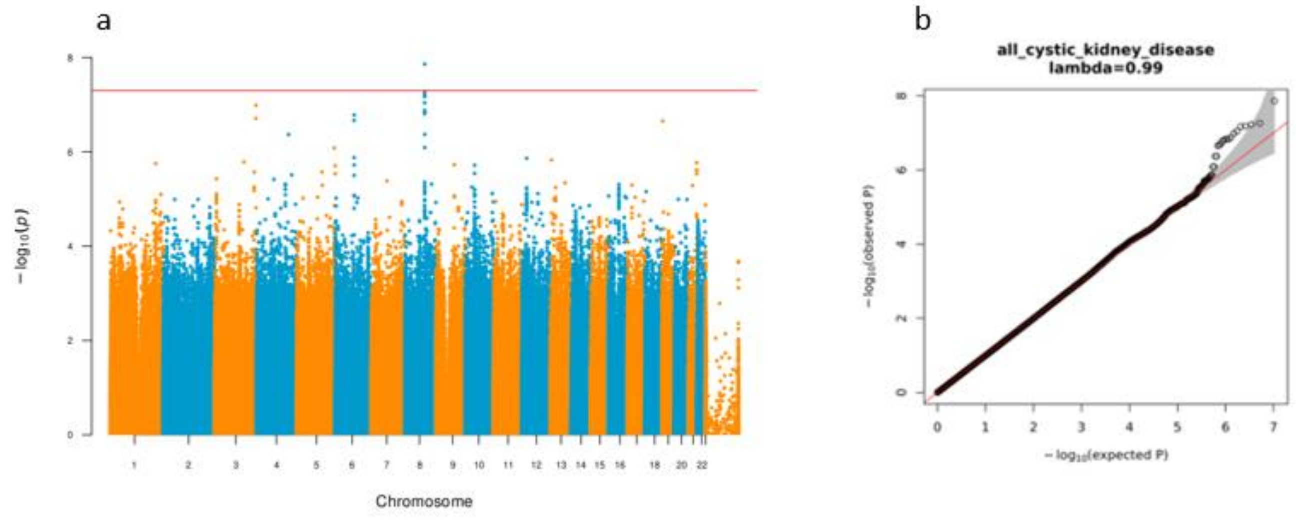

Supplementary Figure S4 – 100KGP CyKD GWAS

3a seqGWAS Manhattan plot of 1209 cystic kidney disease cases against 26096 ancestry matched controls. A single variant reached genome-wide significance chr8:92259567:A:C (P=1.38x10-8, OR 0.72). 3b – Quantile-Quantile plot of the association test in 3a. The genomic inflation is 0.99.

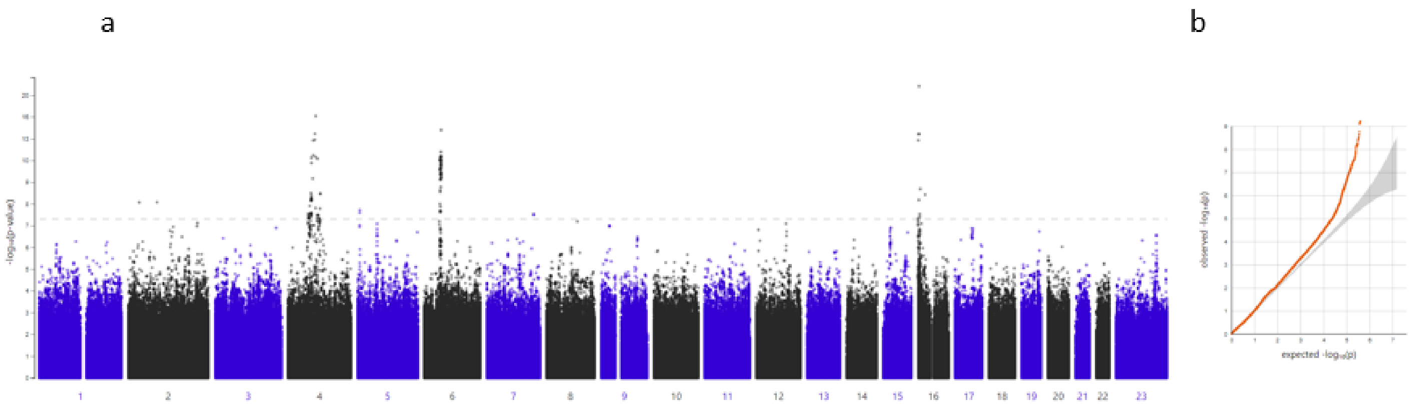

Supplementary Figure S5 – Finngen CyKD GWAS

4a GWAS Manhattan from Finngen plot of 780 cystic kidney disease cases against 375708 controls. 4b – Quantile-Quantile plot of the association test in 3a. The genomic inflation is 1.04.

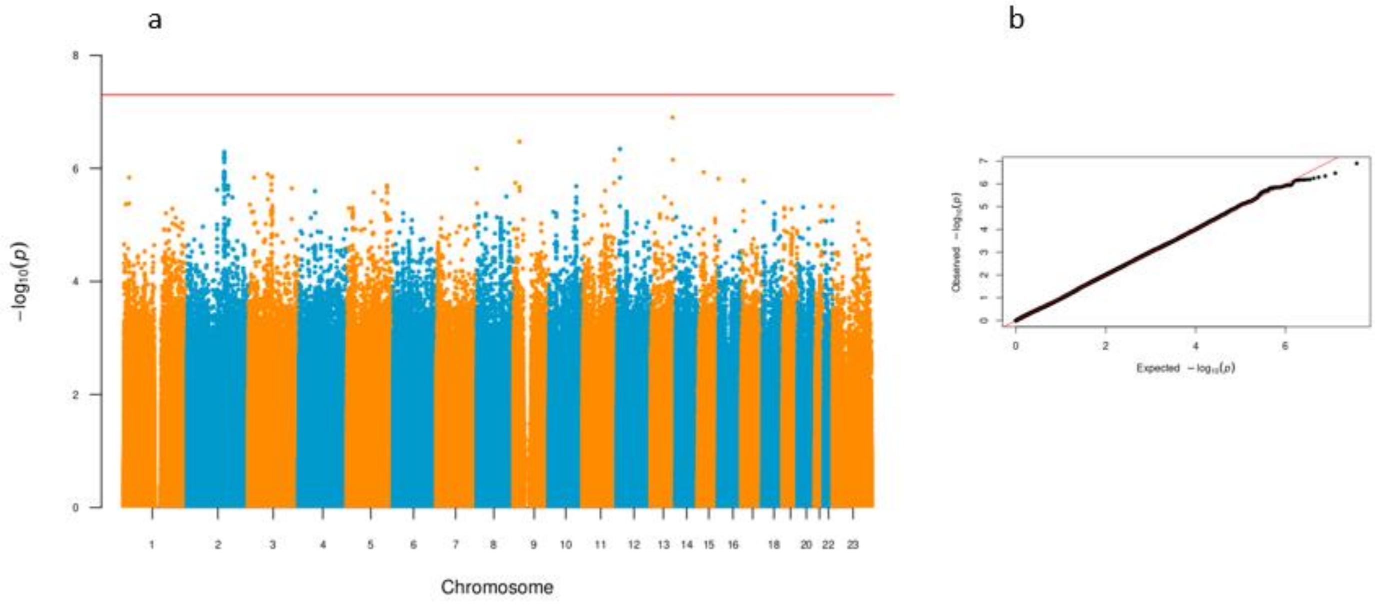

Supplementary Figure S6 – UKBB/JBB CyKD GWAS

5a GWAS Manhattan from UKBB/JBB analysis - plot of 932 cystic kidney disease cases against 534581 controls. 5b – Quantile-Quantile plot of the association test in 5a. The genomic inflation is 1.02.

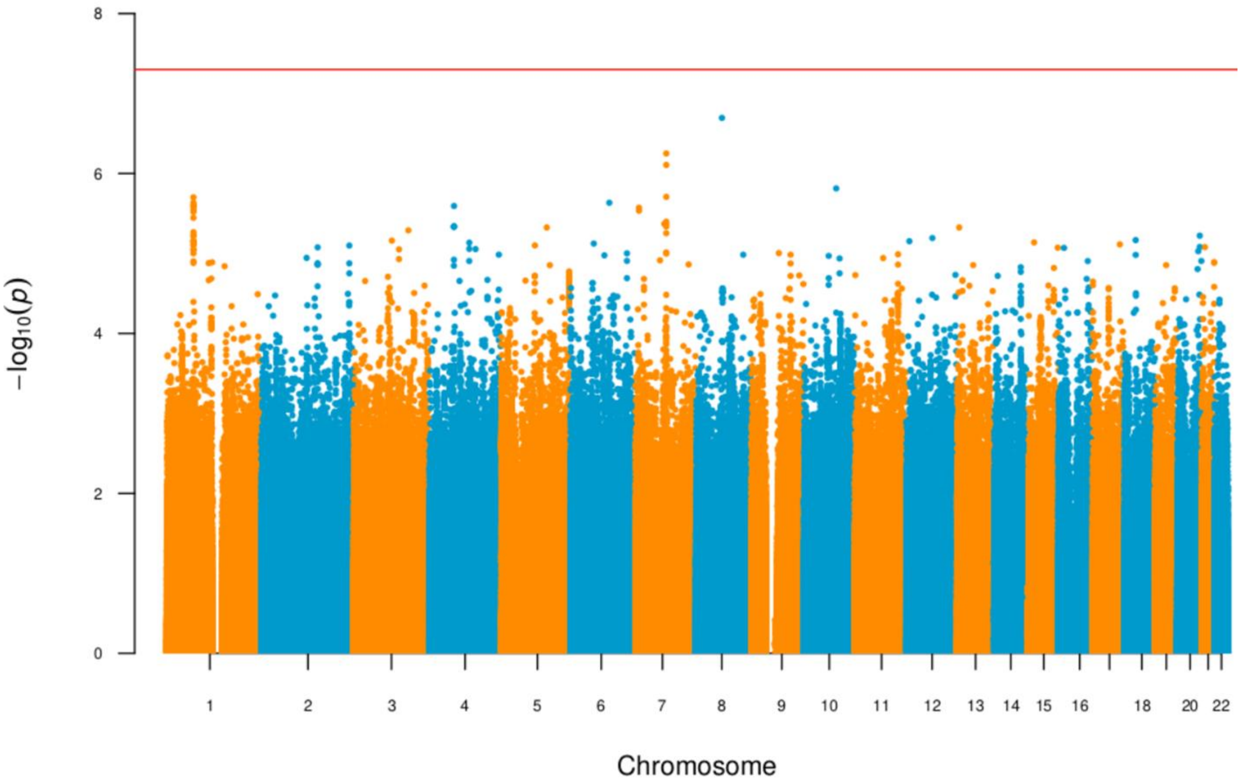

Supplementary Figure S7 - Metanalysis of CyKD GWAS reveals no replicable common variant associations.

Manhattan plot of CyKD metanalysis across 6,641,351 markers in 2,923 cases (1,209 GEL, 780 FinnGen, 510 JBB, 424 UKBB) and 900,824 controls (26,096 GEL, 341,081 FinnGen, 178,216 JBB, 355,431 UKBB). There is no variant that reaches genome-wide significance. The genomic inflation is 1.012.

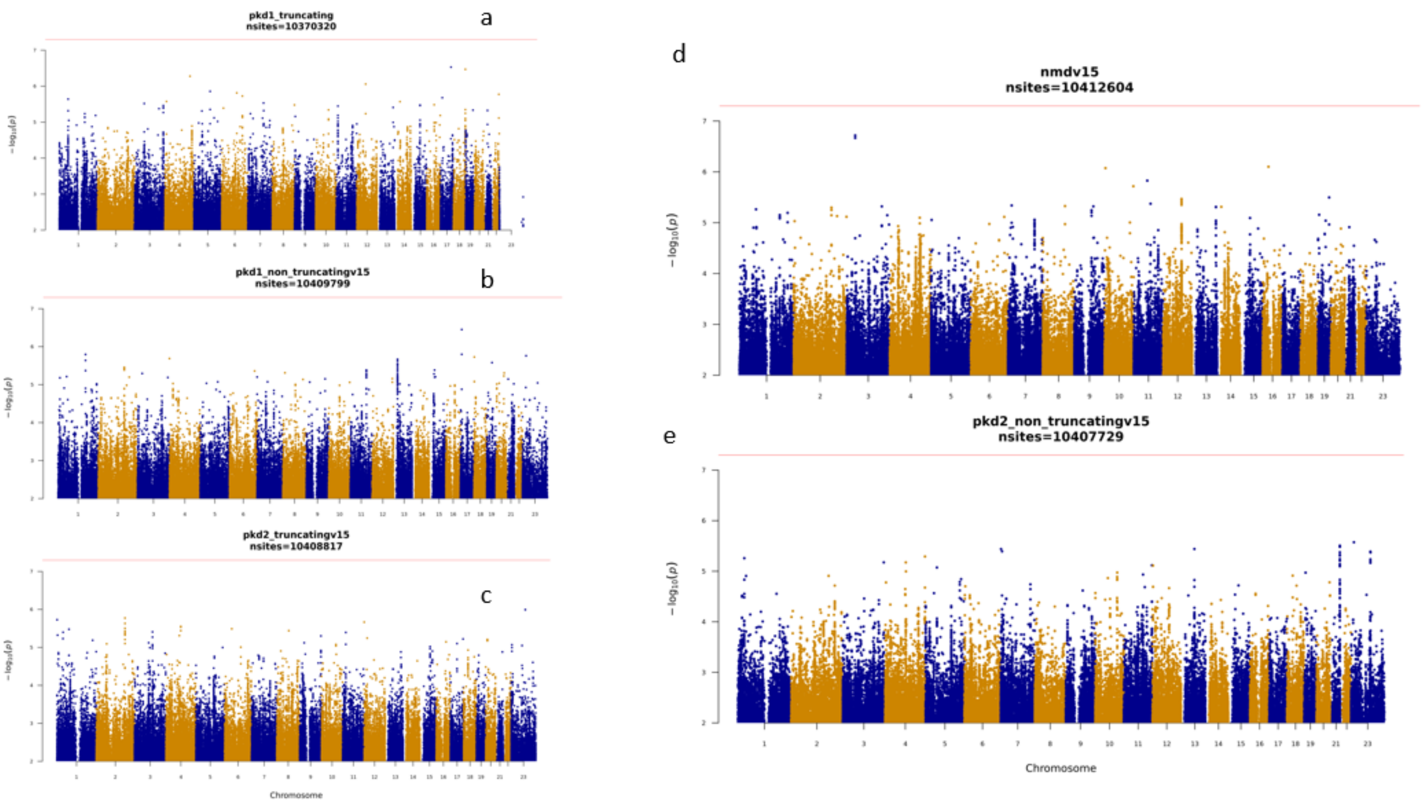

Supplementary Figure S8 - Manhattan plots of GWAS by primary driving variant

SeqGWAS Manhattan plot of cystic kidney disease cases against 26096 ancestry matched controls by primary variant type. 6a – PKD1 truncating, 6b – PKD1 non truncating, 6c - PKD2 truncating, 6d - No primary variant detected, 6e – PKD2 non truncating. There is no variant that reaches genome wide significance in any analysis.

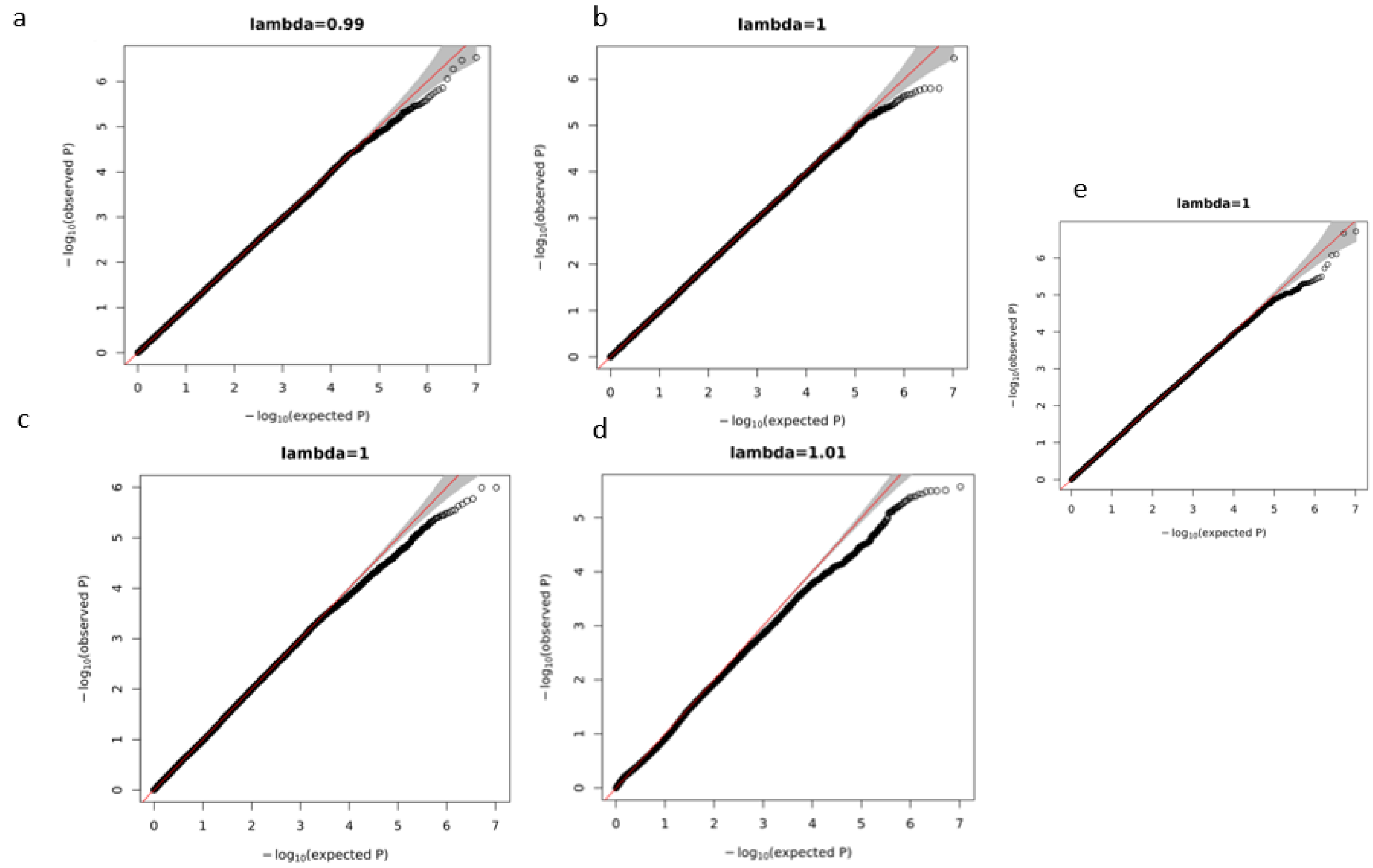

Supplementary Figure S9 - QQ plots of primary variant GWAS

QQ plots SeqGWAS per primary 7a – PKD1 truncating, 7b – PKD1 non truncating, 7c - PKD2 truncating, 7d -PKD2 non truncating, 7e – No primary variant detected. There is no evidence of genomic inflation in the mixed ancestry cohort.

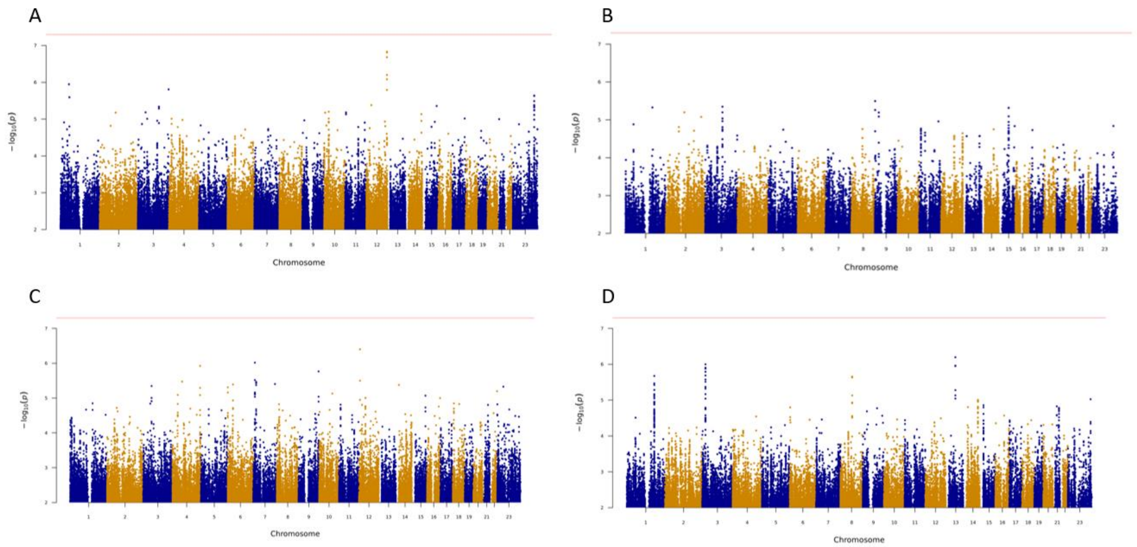

Supplementary Figure S10 - Manhattan plots of time-to-event GWAS in the total CyKD and primary driving variant cohorts.

Manhattan plot of the time-to-event GWAS across the total CyKD cohort and divided by primary variant and no variant detected cohorts. A – Total CyKD cohort (11485299 markers), B – No variant detected cohort (7718522 markers), C – PKD1-truncating cohort (8543817 markers), D – PKD1-nontruncating cohort (7465234 markers). The red line represents genome wide significance.   There were too few events for PKD2 to be analysed.

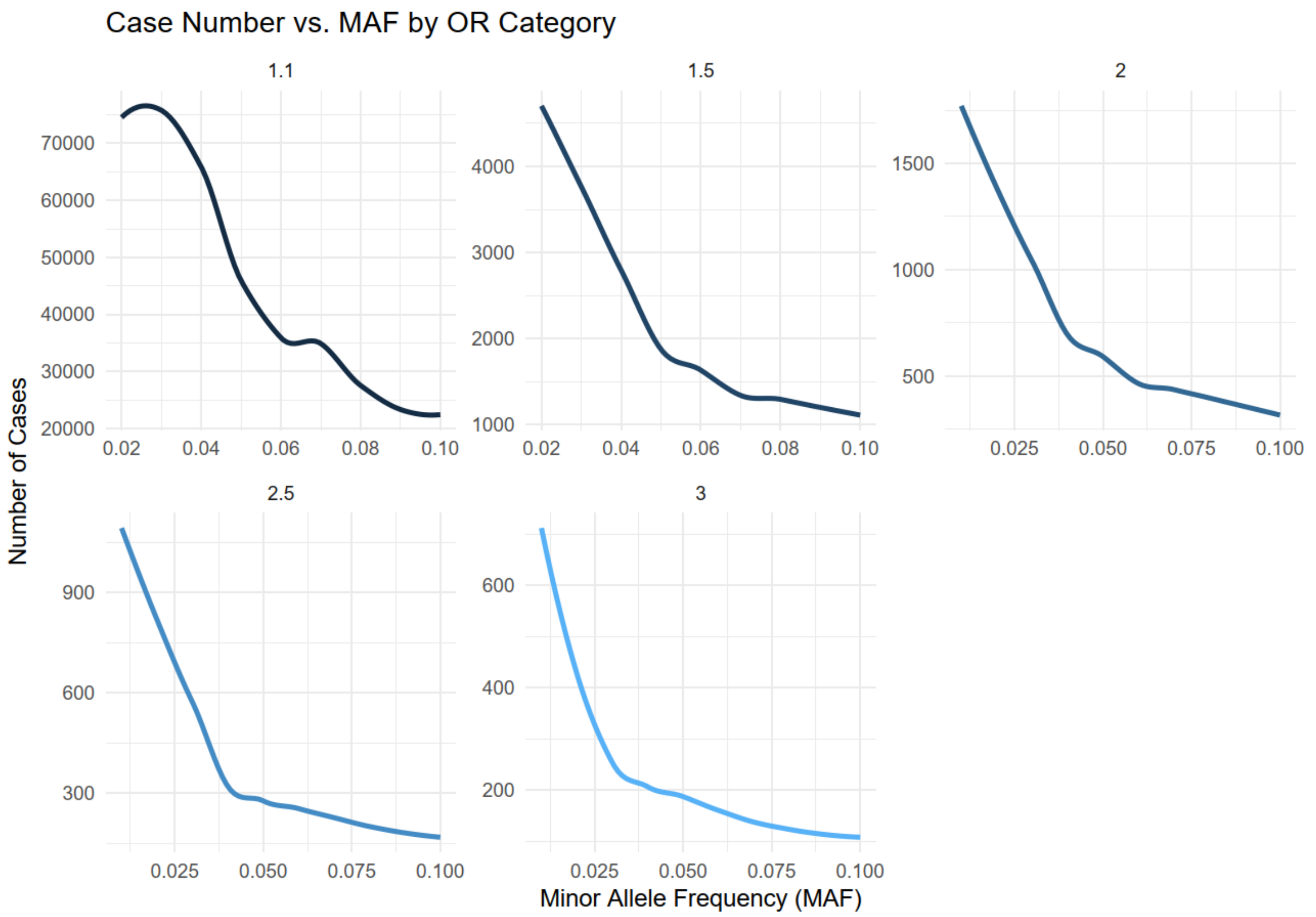

Supplementary Figure S11 – Power calculations for the CyKD GWAS

Plot representing the case numbers against the MAF per OR category to detect a signal in a CyKD GWAS assuming a case rate of 1:1000 at a power of 80% with a genome wide significance of $5x10^{-08}$ *under an additive model.*

References

1. Richards S, Aziz N, Bale S, Bick D, Das S, Gastier-Foster J, et al. Standards and guidelines for the interpretation of sequence variants: a joint consensus recommendation of the American College of Medical Genetics and Genomics and the Association for Molecular Pathology. Genet Med. 2015 May 8;17(5):405–24.

2. Chan MMY, Sadeghi-Alavijeh O, Lopes FM, Hilger AC, Stanescu HC, Voinescu CD, et al. Diverse ancestry whole-genome sequencing association study identifies TBX5 and PTK7 as susceptibility genes for posterior urethral valves. Elife [Internet]. 2022 Sep 1;11. Available from: https://pubmed.ncbi.nlm.nih.gov/36124557/

3. Rimmer A, Phan H, Mathieson I, Iqbal Z, Twigg SRF, WGS500 Consortium, et al. Integrating mapping-, assembly- and haplotype-based approaches for calling variants in clinical sequencing applications. Nat Genet. 2014 Aug;46(8):912–8.

4. Lander ES, Linton LM, Birren B, Nusbaum C, Zody MC, Baldwin J, et al. Initial sequencing and analysis of the human genome. Nature. 2001 Feb 15;409(6822):860–921.

5. Tan A, Abecasis GR, Kang HM. Unified representation of genetic variants. Bioinformatics. 2015 Jul 1;31(13):2202–4.

6. McLaren W, Gil L, Hunt SE, Riat HS, Ritchie GRS, Thormann A, et al. The Ensembl Variant Effect Predictor. Genome Biol. 2016 Jun 6;17(1):1–14.

7. Rentzsch P, Witten D, Cooper GM, Shendure J, Kircher M. CADD: predicting the deleteriousness of variants throughout the human genome. Nucleic Acids Res. 2019 Jan 8;47(D1):D886–94.

8. Karczewski KJ, Francioli LC, Tiao G, Cummings BB, Alföldi J, Wang Q, et al. The mutational constraint spectrum quantified from variation in 141,456 humans. Nature. 2020 May 27;581(7809):434–43.

9. Taliun D, Harris DN, Kessler MD, Carlson J, Szpiech ZA, Torres R, et al. Sequencing of 53,831 diverse genomes from the NHLBI TOPMed Program [Internet]. bioRxiv. bioRxiv; 2019. Available from: http://dx.doi.org/10.1101/563866

10. Danecek P, Bonfield JK, Liddle J, Marshall J, Ohan V, Pollard MO, et al. Twelve years of SAMtools and BCFtools. Gigascience [Internet]. 2021 Feb 16;10(2). Available from: https://pubmed.ncbi.nlm.nih.gov/33590861/

11. Purcell S, Neale B, Todd-Brown K, Thomas L, Ferreira MAR, Bender D, et al. PLINK: a tool set for whole-genome association and population-based linkage analyses. Am J Hum Genet. 2007;81(3):559–75.

12. Manichaikul A, Mychaleckyj JC, Rich SS, Daly K, Sale M, Chen WM. Robust relationship inference in genome-wide association studies. Bioinformatics. 2010 Nov 11;26(22):2867.

13. Privé F, Aschard H, Ziyatdinov A, Blum MGB. Efficient analysis of large-scale genome-wide data with two R packages: bigstatsr and bigsnpr. Bioinformatics. 2018 Aug 15;34(16):2781–7.

14. Privé F. Using the UK Biobank as a global reference of worldwide populations: application to measuring ancestry diversity from GWAS summary statistics. Bioinformatics. 2022 Jun 27;38(13):3477–80.

15. Kurki MI, Karjalainen J, Palta P, Sipilä TP, Kristiansson K, Donner K, et al. FinnGen: Unique genetic insights from combining isolated population and national health register data [Internet]. bioRxiv. 2022. Available from: https://www.medrxiv.org/content/10.1101/2022.03.03.22271360v1.abstract

16. Sakaue S, Kanai M, Tanigawa Y, Karjalainen J, Kurki M, Koshiba S, et al. A cross-population atlas of genetic associations for 220 human phenotypes. Nat Genet. 2021 Sep 30;53(10):1415–24.

17. Mbatchou J, Barnard L, Backman J, Marcketta A, Kosmicki JA, Ziyatdinov A, et al. Computationally efficient whole-genome regression for quantitative and binary traits. Nat Genet. 2021 Jul;53(7):1097–103.

18. Lee SH, Wray NR, Goddard ME, Visscher PM. Estimating missing heritability for disease from genome-wide association studies. Am J Hum Genet. 2011 Mar 11;88(3):294–305.

19. Ioannidis NM, Rothstein JH, Pejaver V, Middha S, McDonnell SK, Baheti S, et al. REVEL: An Ensemble Method for Predicting the Pathogenicity of Rare Missense Variants. Am J Hum Genet. 2016 Oct 6;99(4):877–85.

20. Zhou W, Bi W, Zhao Z, Dey KK, Jagadeesh KA, Karczewski KJ, et al. SAIGE-GENE+ improves the efficiency and accuracy of set-based rare variant association tests. Nat Genet. 2022 Sep 22;54(10):1466–9.

21. Lee S, Emond MJ, Bamshad MJ, Barnes KC, Rieder MJ, Nickerson DA, et al. Optimal unified approach for rare-variant association testing with application to small-sample case-control whole-exome sequencing studies. Am J Hum Genet. 2012 Aug 10;91(2):224–37.
